# Cross-Talk of the CNS With Immune Cells and Functions in Health and Disease

**DOI:** 10.3389/fneur.2021.672455

**Published:** 2021-05-31

**Authors:** Agata Matejuk, Arthur A. Vandenbark, Halina Offner

**Affiliations:** ^1^Department of Immunology, Collegium Medicum, University of Zielona Góra, Zielona Góra, Poland; ^2^Neuroimmunology Research, VA Portland Health Care System, Portland, OR, United States; ^3^Department of Neurology, Oregon Health and Science University, Portland, OR, United States; ^4^Department of Molecular Microbiology and Immunology, Oregon Health and Science University, Portland, OR, United States; ^5^Department of Anesthesiology and Perioperative Medicine, Oregon Health and Science University, Portland, OR, United States

**Keywords:** innate and adaptive immunity, CNS, microglia, oligodendrocytes, neuroinflammation

## Abstract

The immune system's role is much more than merely recognizing self vs. non-self and involves maintaining homeostasis and integrity of the organism starting from early development to ensure proper organ function later in life. Unlike other systems, the central nervous system (CNS) is separated from the peripheral immune machinery that, for decades, has been envisioned almost entirely as detrimental to the nervous system. New research changes this view and shows that blood-borne immune cells (both adaptive and innate) can provide homeostatic support to the CNS via neuroimmune communication. Neurodegeneration is mostly viewed through the lens of the resident brain immune populations with little attention to peripheral circulation. For example, cognition declines with impairment of peripheral adaptive immunity but not with the removal of microglia. Therapeutic failures of agents targeting the neuroinflammation framework (inhibiting immune response), especially in neurodegenerative disorders, call for a reconsideration of immune response contributions. It is crucial to understand cross-talk between the CNS and the immune system in health and disease to decipher neurodestructive and neuroprotective immune mechanisms for more efficient therapeutic strategies.

## Introduction

The immune system protects us from all possible threats that could endanger the homeostasis of the body. The primary two arms of this protective system are innate and adaptive immunity. Both are represented by specific cells that directly (cell-to-cell) or indirectly (via mediators) eliminate danger. The innate system is older phylogenetically as compared to adaptive, exists in most life forms, is rapid and non-specific, and is highly efficient right at birth. Adaptive immunity is present only in jawed vertebrates, matures over time, is specific, and creates memory cells that can protect the organism throughout life (1). Regarding the central nervous system (CNS), immune responses are complex and unique, and so far, mechanisms operating in different disorders are poorly understood. This directly translates to poor treatment efficacies. Classic immune responses in the CNS relate to infectious or autoimmune diseases with T and B cells as leading players and tissue damage caused by inflammatory infiltrates (2). In inflammatory brain diseases such as bacterial meningitis and encephalitis, the primary immune response is initiated by meningeal macrophages activated by proinflammatory mediators from lysed bacteria (3). In the case of neurodegeneration or metabolic disorders, immune responses depend on innate immune activation with the involvement of microglia, macrophages, and astrocytes (4). Independent of the initiating machinery, after the insult, immune-competent cells are programmed to dampen the response and start tissue repair and functional healing. Mechanisms that are responsible for the vicious inflammatory loop that turns into chronic disease are not well-understood. One possible mechanism is based on persistent systemic inflammation coming from the periphery that can permanently change cognitive and behavioral states and lead to neurodegenerative disorders (5). It is well-known that cognitive decline is common in sepsis survivors, as well as patients suffering from chronic inflammatory conditions. Almost all systemic lupus erythematosus (SLE) patients develop neuropsychiatric dysfunction.

Cells that define the CNS and its neurological functions are neurons, which constitute only about 1/3 of the whole brain volume. The remainder are glial cells such as microglia, astrocytes, and oligodendrocyte lineage cells. Their central role is to create a neuron-supportive environment. Astrocytes possess a wide range of homeostatic functions including metabolic support and synaptic connections coordination. Microglia during development remove apoptotic debris, ensure synapse formation, influence neuronal survival and modulate vascularization and in the adult, survey the parenchymal environment, phagocytose synaptic rudiments, and modulate neuroplasticity. Oligodendrocyte lineage cells are responsible for myelinization and metabolic support for axons, form synapses with neurons, and sustain the blood–brain barrier (BBB) integrity. All glial cells can be classified as immune-competent cells within the CNS and possess characteristics of innate immunity. They respond to neuronal injury with programs that include propagation, changes in morphology, production of inflammatory molecules, and debris clearing. The brain is separated from the rest of the body by the BBB, which blocks passage of cells including immune cells and many molecules including antibodies. In this respect, the brain received the status of an immune-privileged organ. First studies that led to this concept came from the 19th-century experiments with dyes that stained all tissues except the brain, suggesting that the brain is efficiently separated from the rest of the body. Discovery of the BBB as a shielded structure that keeps away peripheral immune cells and mediators, the absence of classical antigen-presenting cells in CNS, diminished expression of MHC molecules, and lack of the classic lymphatic system further strengthened the concept of the CNS as an immunologically barricaded organ (6). However, pioneering work by Medawar in 1948 questioned this concept showing the existence of an immune response to CNS antigens (7). Gradually, the viewpoint of the CNS as an immune-privilege site became questioned (8, 9). Discoveries based on studies using cell-specific targeting, *in vivo* imaging, and single-cell expression analysis revealed the existence of meningeal lymphatic vessels and opened new perceptions of the interaction between CNS and the immune system (10, 11). Indeed, the CNS has now been suggested to be an actively regulated site of immune surveillance where the exchange between the brain and the periphery is a dynamic process necessary for the well-being of the entire nervous system (12). Moreover, the peripheral immune system, especially the adaptive arm often considered to be harmful, might be an essential guardian for CNS health.

It is crucial to realize that in the brain, the function of immune sentinels extends far beyond their classic roles in the peripheral immune system. Several cytokines, including IL-1β, IL-6, IL-10, IFNs, TNF-α, and TGF-β, have been observed in the healthy brain where they perform a wide range of functions (13). Additionally, direct translations of cytokine functions that are well-defined in the periphery may not operate similarly within CNS context, such as TNF-α displaying neuroprotective properties or TGF-β1 that is upregulated in aging and after CNS injury (14, 15) as well as in EAE, a mouse model for multiple sclerosis (MS) (16). Cytokines, pleiotropic proteins, and chemokines with established roles in systemic immunity are crucial during brain development. Cytokines play a role as neurotrophic factors, promote survival or apoptosis of cells, and facilitate proper inter-neuronal connections. Chemokines guide neurons and glia to the proper location. In the inflammatory brain, the role of cytokines that exhibit extraordinary functional redundancy with therapeutic potential is broadly described elsewhere (17, 18). One example is the IL-12 family of heterodimeric cytokines with a remarkable influence on innate and adaptive immune responses. One of the IL-12 family members, IL-35, is a potent inducer of regulatory B cells able to augment CNS autoimmunity (17). An important aspect that links the immune system with the nervous system is that immune cells express adrenoreceptors responsive to noradrenaline and adrenaline, resulting in anti-inflammatory effects (19). Downregulation of these receptors on microglia and astrocytes may contribute to autoimmunity or neurodegeneration. Recently, extracellular vesicles (EVs) as a newly recognized means of cell-to-cell communication, not only among CNS cells but also between CNS and the periphery, have been extensively studied and described (20). EVs secreted by almost all cell types are cargos filed with bioactive molecules such as proteins including cytokines, chemokines, neurotransmitters, and nucleic acids including DNA, RNA, and miRNA. Although current findings in the EV field need further exploration, like, for example, the mechanisms by which peripheral EVs cross the BBB and contribute to neuroinflammation and *vice versa*, they can play an important role in the diagnosis and as new therapeutics of CNS disorders (21).

Close communication between the immune system and the CNS is evident, and any dysfunction within the immune machinery may lead to neurological disorders. In this review, we present recent findings highlighting the role of innate immune responses mostly operating within the CNS and adaptive immune responses in brain health and disease with a specific focus on MS and Alzheimer's disease (AD).

## Immune Components in Brain Development

The involvement of immune mediators, especially cytokines and chemokines, in brain development starts right at the beginning and includes coordinating trafficking, proliferation, and differentiation of neuronal and glial cells.

### Neurogenesis Is Synchronized With Microglia Development

As early as day 19 after conception, human hematopoiesis starts to proceed within the yolk sac, creating erythromyeloid precursors for yolk sac macrophages, which further colonize the brain and become microglia cells. Microglia development is synchronized with neurogenesis. Neurogenesis happens under the supervision of the TGF-β cytokine superfamily, especially bone morphogenic proteins (BMPs), which repress the induction of the process. Additionally, in the embryonic brain, many gp130 family cytokines are present such as LIF, CNTF, CT-1, and NP, where they play a role in the neuroepithelial/radial glial cell (RGC) self-renewal (22). The role of the gp130 family of cytokines, also referred to as a neuropoietic family, in nervous system development, disease, and injury, is broadly described in a review paper by Bauer et al. (23). RGCs create a pool of precursor cells for neurons, astrocytes, oligodendrocytes, and scaffolds for radially migrating neurons. Embryonic progenitor proliferation depends on IL-1β, a cytokine that induces neuronal stem and progenitor cell proliferation in adults as a response to injury and disease (24). During brain development and in the adult brain in mice, the microglia proliferation, differentiation and survival, and maintenance critically depend on CSF-1R (25) and its ligands CSF1 and IL-34. Blockage of CSF-1R leads to microglia elimination and abnormal circuit connectivity in adult mice (26). IL-34 is the cytokine that controls the growth and development of myeloid cells and in the brain is produced by neurons. Both CSF1 and IL-34 are essential for proper microglia development in mice (27). Further steps of microglia development, including differentiation, molecular, and functional identity, depend on TGF-β released in the brain, mostly by astrocytes (28). As demonstrated by experiments in mouse models, mice lacking TGFβR2 were characterized by an activated phenotype (29). It has been proposed that disturbances within the TGF-β signaling may play a role in pathological conditions of the CNS (30). Interestingly, Wlodarczyk et al. discovered a functionally diverse type of microglia in neonatal brain participating in CNS myelination. This new transient subset of cells was characterized by expression of CD11c and high levels of IGF1 mRNA that might directly influence myelination by stimulating oligodendrocytes (31). Furthermore, a study by Hagemeyer et al. revealed the existence of a distinct, early postnatal microglia population in mouse white matter that regulates myelinogenesis by shaping the number of oligodendrocyte progenitor cells (32). This active interaction between microglia and oligodendrocyte progenitor cells was also evident in the adult brain. As recently shown by Lloyd et al., an efficient remyelination later in life in mice depends on microglia necroptosis and transition to a pro-regenerative state (33).

### Synaptic Pruning Critically Depend on Complement Compounds

In the developing brain, microglia are involved in neurogenesis and synaptic pruning. Microglia actively eliminate and phagocytose neurons via the production of toxic products such as superoxide ions, nerve growth factor (NGF), and TNF, which induce apoptosis without inflammation triggered by CR3/DAP12 signal transduction (34). Microglial phagocytic activity during development is also maintained by TREM 2. Mice lacking this microglia receptor display increased synaptic density and poor learning and social behavior in adulthood (35). Phagocytosis is linked to apoptosis through signals like ATP released by apoptotic cells (36). Classical complement cascade plays a crucial role in the process of developmental synaptic pruning. Synapses destined for removal are tagged by complement components such as C3 and C1q and recognized in this form by microglia for elimination. Moreover, microglia are the main sources for C1q and CR5 (37). Aberrant pruning and excessive synaptic loss has been observed in several neurological and psychiatric conditions. Re-activation of complement-mediated synaptic elimination is involved in cognitive decline in AD (38) and virus-mediated memory impairment (39). As presented by human genetic studies, higher levels of C4a expression have been linked to increased risk for schizophrenia (40). An important contribution to synaptic elimination and maturation during early postnatal period is attributed to CX3CL1–CX3CR1 interaction (41). Abnormal synaptic pruning by microglia in a complement-dependent manner and/or via fractalkine-receptor signaling leads to wiring anomalies (41, 42). Some developmental processes based on complement-dependent phagocytosis by microglia may occur in a gender-specific manner, as shown in a rat model (43).

### Astrocytes Control Synaptogenesis

Synaptogenesis coincides with astrogenesis, and as shown by experiments with neuronal cell culture, the survival of isolated neurons and formation of synapses is dependent on astrocytes and/or their factors (44). An astrocyte's crucial role in brain development is an involvement in synaptogenesis and regulation of neuronal circuits by direct cell-to-cell contact and by soluble mediators. Direct contact is maintained by adhesion molecules present on neurons and astrocytes, and these play a vital role during the embryonic formation of excitatory and inhibitory synapses. Direct neuron–astrocyte contact mediated by neurexin–neuroglin cross-talk not only facilitates neuronal synaptic function but also supports astrocytic morphology (45). Astrocytes secrete both stimulatory and inhibitory mediators associated with synaptogenesis, thus possessing effective control over the creation and development of synapses. Some astrocytic factors that control synaptogenesis, the formation of structural synapses, regulation of synaptic plasticity, and postsynaptic receptor levels are Secreted Protein Acidic, Rich in Cysteine (SPARC), thrombospondin (TSP1,2), hevin, and glypicans. The broad aspects of the biology of astrocyte–synapse interactions are reviewed elsewhere (46, 47). The same crucial role in the modulation of postsynaptic receptor levels is attributed to immune mediators such as TNF-α. This cytokine induces active synapses by increasing AMPA receptors and reducing GABA receptors present in inhibitory synapses. Besides secreting synaptogenic modulators shaping circuit development, astrocytes respond and get activated by signals coming from neurons by virtue of neurotransmitter receptors' expression (48). Astrocytes respond to neuronal mediators by elevating calcium levels (49), leading to the release of gliotransmitters such as ATP, GABA, and glutamate that modulate astrocytic and neuronal activity (50). The presynaptic terminal development and function *in vivo* as well as regulation of synaptic transmission is partially dependent on cholesterol also synthesized by astrocytes. Mice with interrupted lipid synthesis show lessened synaptic development and plasticity (51). These findings establish a critical role for astrocytic lipid metabolism. During neurodevelopment, astrocytes' final pruning of synapses is performed on a much higher level than microglia. Astrocytic direct engulfment of synaptic material is maintained through MEGF10 and MERTK phagocytic pathways (52). In mice, an indirect pathway is based on the release of TGF-β, which increases complement C1q expression in neurons and makes them visible for phagocytosis by microglia (53). Another way of indirect phagocytosis is the release of astrocytic IL-33, a member of the IL-1 family, which sends signals to phagocytic microglia via the IL1RL1 receptor (54).

The deep understanding of active pathways during development associated with synaptic pruning or eliminating neurons is of key importance, as some of these pathways may be reactivated during neurodegenerative diseases such as AD. The triparty axis composed of complement–microglia–astrocytes is especially important in synapsis elimination during development. In adulthood, the complement C1q is largely downregulated. In many neurodegenerative disorders, the complement cascade may become re-activated (55). Recently, in a mouse model, re-activation of complement C1q early in AD leading to synaptic loss by microglia, has been shown even before plaque deposition (38). In addition, it is possible that some populations of microglia, which are physiologically present during development and are inactivated during adulthood, may appear in pathological conditions. A better understanding of the role of such populations may allow for a better understanding of neuropathology.

## An Innate Arm Of Immunity in Brain Health

**Figure 1:**
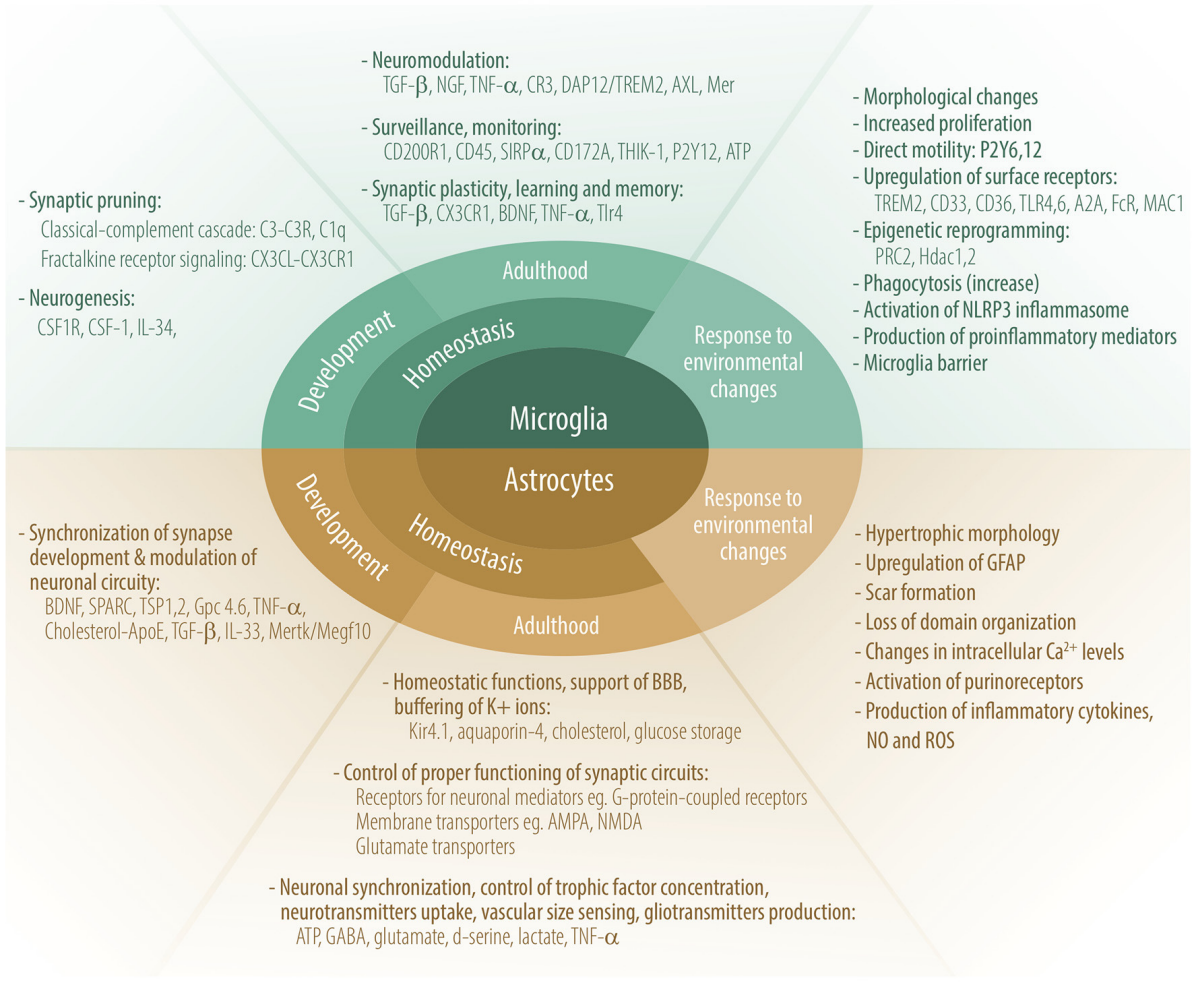
Role of microglia and astrocytes in homeostasis during CNS development and adulthood, and their main responses to environmental changes. Some molecules participating in these processes are depicted.

Common knowledge is that the CNS lacks a wide array of immune sentinels with only one population of immune-competent cells—microglia. However, recent advances in neuroimmunology research shed new light on immune-competent cells in the brain. Besides microglia, astrocytes, oligodendrocytes, and non-parenchymal macrophages also possess characteristics linked to innate immunity such as phagocytosis, antigen presentation, and expression of innate-immunity sensors (TLRs, NODs), and the production and response to cytokines. Of note, as innate-immune cells, microglia and astrocytes produce significant amounts of antimicrobial peptides (AMP) that provide immune surveillance against pathogens, regulate the immune response and inflammation within the CNS, and maintain a healthy tissue (56, 57). Cross-talk between microglia and astrocytes is fundamental for brain homeostasis (58). The role of microglia and astrocytes in homeostasis during CNS development and adulthood, and their main responses to environmental changes are shown in Figure 1.

### Microglia, the Primary Myeloid Cells in The Brain

Brain macrophages comprise four distinct cell populations: microglia, meningeal, perivascular, and choroid plexus macrophages. Microglia are the only parenchymal macrophages, while other macrophages occupy most strategic frontiers between the parenchyma and vasculature (59). Microglia are derived from yolk sac (YS) erythro-myeloid progenitors that migrate to the developing brain before BBB is formed (25). Microglia and macrophages in the peripheral immune system come from the same primitive progenitor; however, early during embryonic life, their developmental pathways separate. Microglia populate the CNS parenchyma, creating a long-lived pool of cells that renew independently from hematopoietic cells. Meningeal and perivascular macrophages also come from YS precursors, whereas choroid plexus macrophages have both embryonic and adult hematopoietic ontogeny (60). Thus, only choroid plexus macrophages can be replenished by macrophages from the circulation. The others are maintained by self-renewal. Any insult to CNS may result in the recruitment of bone marrow-derived myeloid cells and replacement of perivascular macrophages (61). All CNS macrophages create a morphologically, phenotypically, and functionally heterogeneous system, occupy specific localizations, and cover the entire CNS (59, 60). Each population's exact function is still poorly understood, mostly because of the significant research burden arising from overlapping combinations of phenotypic and genetic characteristics among populations. Microglia differ from other brain macrophages by a lower expression of MHC II and CD45. Their genetic and phenotypic characteristics resemble more of brain non-parenchymal macrophages than blood-borne myeloid cells. Genes such as Tmem119, P2ry12, and Sall1 are considered microglia-specific markers (29, 62). It has been found that Sall1 is a key transcription factor in maintaining a quiescent microglia phenotype, and its loss is linked to upregulation of genes found in other tissue macrophages (29). Conversion from microglia to other brain macrophages during development is possible, as shown by Wong et al. in mice (63). By blocking the transmembrane protein negative regulator of reactive oxygen species (NRROS) that promotes microglial identity, microglial specific genes were downregulated, whereas genes characteristic for perivascular macrophages were upregulated (63). Interestingly, in the adult brain, Sall1, not NRROS, is required for microglial identity. In general, true macrophage characteristics are shaped by the local environment and epigenetics (64), and in the case of mouse microglia as shown by cell transplantation systems, the combination of ontogeny and brain environmental cues critically controls microglial uniqueness and their exquisite plasticity (65). A homeostatic signature is imprinted in microglia, which even after excessive *in vitro* manipulations is recovered once microglia are implanted back into adult mouse brain (66). Hematopoietic stem cell–microglia-like cells (HSC-MLC), although able to mimic microglia, have been found to be genetically different and enriched in genes associated with neurological diseases such as AD (65). Different mouse CNS regions are characterized by distinctive subpopulations of microglia with unique transcriptomic profiles, morphology, and function, most pronounced in early development (67). Interestingly, despite the high diversification of microglia subpopulations during early development, a predominance of genes associated with metabolism, growth, motility, and proliferation has been found. Some of those genes can probably be re-activated during injury and in the aging brain. An essential difference between mouse and human microglia is the partially activated phenotype even in the absence of pathology in the human brain (68), where microglia display more complex phenotypes (69). The activated phenotype of the human brain might be associated with the fact that humans are exposed to systemic infections or might be related to age factors like neurodegeneration or diet that can change the gut microbiome's composition. Thus, although useful, animal models do not entirely reflect the situation that prevails in humans (70). Moreover, a classic division of macrophages to M1-like or M2-like phenotypes is no longer accurate for microglia, as evidenced by modern transcriptome sprofiling (71).

### Microglia Are Restless Cells Protecting Brain Parenchyma

Microglia are restless cells that monitor every aspect of the brain environment using fine processes (72) that sense neuronal activity and changes in molecules, including neurotransmitters and ATP. The latter is a main attractant and stimulus of microglia for differentially regulated non-directional and directed motility. It is also the main molecule used by microglia and neurons to communicate. Activated neurons release ATP that signals microglia via purinergic receptors, as shown by using the larval zebrafish model (73). This is a signal for resting microglia to move processes toward targets, surround highly active neurons, and consequently regulate their activity. Additional ways of communication between microglia and the environment are based on activation of ion channels and cell surface receptors (74). Movement of ramified microglia is controlled by potassium and its channel THIK-1 (75), whereas movement of microglia caused by acute injury is performed via the ATP–P2Y12 receptor axis (76). Microglia detect changes in the environment using ~100 genes called “sensome” (77). It contains proteins as diverse as Fc receptors, purinoceptors, pattern-recognition receptors, different CDs, chemokine receptors, and integrins. CD33 and a triggering receptor expressed on myeloid cells 2 (TREM2) are two genes belonging to the microglia sensome that have been found as risk factors for late-onset AD (78). It has been proposed that mediators released by microglia, such as TREM2 or complement components, may function as early biomarkers for CNS pathology or monitor disease progression and therapeutic efficacy like in the case of AD (79). In the brain, microglia participate in neuromodulation, synaptic plasticity, learning, and memory formation. Microglia can regulate those processes by producing inflammatory cytokines such as interleukins or TNF-α. The latter is a key molecule for proper synapse maturation and plasticity as previously discussed for synaptogenesis. Neurons and microglia control each other via fractalkine–CX3CR1 interaction. The only cells responsive to fractalkine in the brain are microglia (78), and their resting state is under the supervision of fractalkine released mainly by neurons and CNS endothelium. Besides, fractalkine saves microglia from apoptosis and facilitates glutamate uptake by astrocytes (78). The lack of CX3CR1 reduces neurogenesis and weakens learning tasks (80). Thus, microglia in the adult brain mostly regulate processes linked to long-term synaptic plasticity and adult neurogenesis, which underline learning and memory abilities, and abnormal levels of TGF-β or CX3CR1 during development can result in aberration in neuroplasticity in adulthood (34).

### Brain Vital Functions Depend on Astrocytic Actions

Astrocytes (from the Greek *astron* means star), the most numerous glial cells, are star-like cells with long processes assembling a dense network with all other CNS cells. In the human brain, one astrocyte makes connections with ~2 million synapses (81). Astrocytic diversity is most pronounced in humans. Human astrocytes are 4 times bigger and possess 10 times more processes than mouse astrocytes (82), features that most likely are responsible for logical thinking and cognitive abilities. Astrocytes, neurons, and oligodendrocytes come from the same progenitor cells called RGCs that originate from neuroepithelial stem cells. Radial cells create the main pool of brain cells and additionally help neurons migrate to their final location by serving as scaffolds (46). Astrogenesis starts during the late stages of neurogenesis, and the whole set of brain astrocytes is created throughout the 1st month of life. Astrocytes form a pool of highly polymorphic cells, especially in the human brain (83). So far, an exclusive astrocyte marker is not known to exist, and for decades, glial fibrillary acidic protein (GFAP) has been used to identify astrocytes in the CNS (84). Based on gene expression profiling, astrocytes are far more diverse than previously anticipated (85). Traditionally, astrocytes have been divided into two groups based on morphology and location. One group is operational in gray matter with bushy processes that stay in close touch with blood vessels via endfeet and endsheet synapses (86, 87). The second group occupies white matter, is fibrous in structure, stays in touch with Ranvier nodes, and supports myelination (46). Astrocytes display a wide array of homeostatic functions, and neuronal existence hinges on astrocytes. Long astrocyte processes penetrate all areas of the brain. Endfeet as a part of BBB and cerebral fluid–brain barrier allow metabolite exchange to nourish the CNS and provide nutrients and oxygen from the blood to neurons. Additionally, astrocytic endfeet control ion and water levels via the potassium channel, Kir4.1, present only in astrocytes. For example, astrocytes buffer K+ ions to prevent neuronal over-excitability. Neurons need a constant high supply of energy to cope with their metabolic demands. Astrocytes maintain metabolic stability of the brain, produce cholesterol, and store glucose in the form of glycogen used for lactate production as an energy source for the CNS (88). One of the most critical roles of astrocytes during development is the control of synaptic formation and elimination that lead to proper synaptic connectivity and, in the adult brain, the proper synaptic transmission and function of synaptic circuits. Astrocytes are part of a so-called tripartite synapse together with presynaptic and postsynaptic structures (89), where they control the clearance of toxic neurotransmitters, e.g., glutamate (90). Glutamate together with ATP, gamma-aminobutyric acid (GABA), d-serine, lactate, and TNF-α creates a pool of astrocytic mediators called gliotransmitters used by astrocytes to control many vital functions and influence plasticity of neurons and their communication with microglia and endothelial cells (90). Activation of astrocytes to produce gliotransmitters is partially regulated by oscillations in Ca2+ levels and purinergic receptors (84). Although there is no strong scientific evidence yet, there is a certain probability that astrocytes' excitability based on calcium transients carries codes and computational properties (91). Glutamate in excess is neurotoxic and is removed from synapses by astrocytes that convert it to glutamine. Glutamine is transferred to neurons for glutamate and GABA production. This process is partly assisted by fractalkine, a chemokine produced by neurons that promotes neuroprotection. Microglia are the only cells in the CNS expressing fractalkine receptors. Glutamate production by astrocytes is partially controlled by TNF-α released by microglia. TNF-α together with hevin and SPARC, is a synapse-modifying factor as described above and is responsible for synaptic plasticity called homeostatic scaling, important for bracing the synapse as a function of neuronal activity (92). Glutamate and TNF-α provide a homeostatic feedback loop to limit excitability. Excessive TNF-α levels caused by promoting massive secretion of glutamate are neurotoxic (93).

### Oligodendrocyte Lineage Cells, the Fourth Strength of The Brain

**Figure 2:**
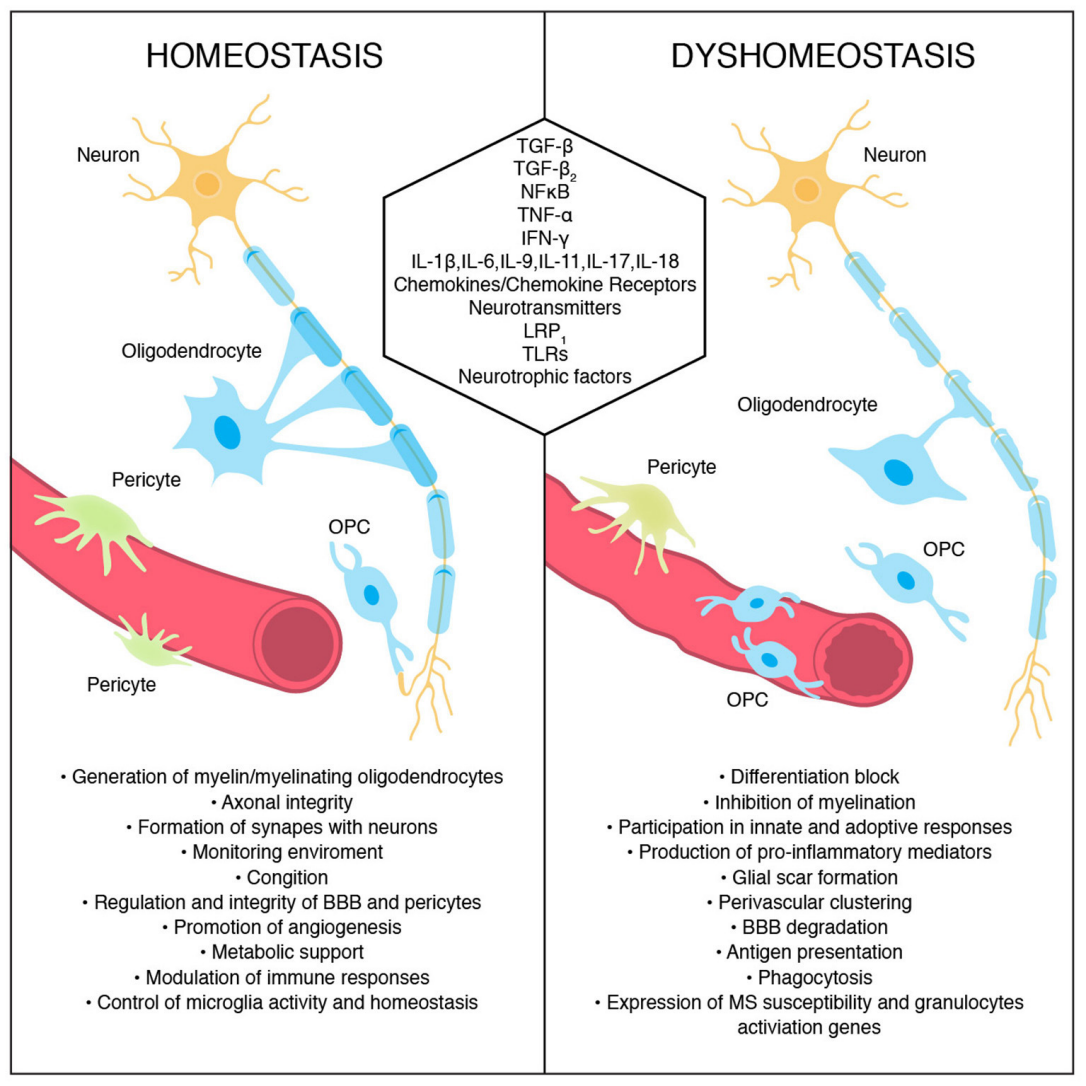
Oligodendrocyte and oligodendrocyte precursors cells' roles during homeostasis and their conduct during dyshomeostasis. Some signals/molecules participating in these processes are presented.

Oligodendrocytes produce myelin sheaths that wrap neuronal axons and sustain axonal integrity and function. Proper myelin insulation allows for undisrupted information passage within neuronal network on which cognitive and motor functions hinge. During embryogenesis, oligodendrocytes are the last brain cells to develop. They arise from oligodendrocyte progenitor cells (OPCs) generated from radial cells in three waves in mice, and in humans around the embryonic–fetal transition (94). OPCs express NG2 chondroitin sulfate proteoglycan and platelet-derived growth factor receptor α (PDGFRα). The latter is a commonly used OPC marker important for proliferation, migration, and, potentially, differentiation. However, those markers are not exclusively expressed by oligodendrocyte lineage cells. OPCs are precursor cells not only for oligodendrocytes but also for Schwann cells, and astrocytes expressing GFAP and S100b (94). One of the key OPC factors in transitioning from immature to myelinating oligodendrocytes is TGF-β (95), which additionally supports BBB integrity (96). Cytokines such as IL-9, IFN-γ and TNF-α inhibit differentiation of OPCs into myelinating oligodendrocytes whereas cytokines like IL-11 and IL-17A enhance OPCs survival and transition to mature oligodendrocytes (97–99). OPCs expressing NG2 create a fourth large population within the glial pool of brain cells (100), are as numerous as astrocytes, and are present within the gray and white matter, neurogenic niche, and optic nerve in the adult CNS (101). In physiological conditions in adult brain, they are responsible not only for generation of oligodendrocytes producing myelin but also for axonal integrity, cognition abilities, and immune responses (102). Although still under discussion, additionally, OPCs seem to participate in diverse processes such as monitoring environment to ensure brain homeostasis, forming functional synapses with neurons, promoting angiogenesis, regulating tightness and function of BBB and pericytes, and providing metabolic support (94). Recently, Zhang et al. showed that NG2^+^ cells are the main producers of TGFα-2, a cytokine that controls microglia activity via CX3CR1 and is responsible for the maintenance of microglia homeostasis under physiological conditions (103). The lack of quiescent NG2^+^ glia but not mature oligodendrocytes in animal models disrupted the resting state of microglia and moderately increased expression of TNF-α suggesting a novel function for NG2^+^ cells in buffering and controlling the immune responses in adult brain. Studies indicate that NG2 controls brain cell activity and may represent a universal tool of maintaining the subtle immune balance across the entire CNS. The functions of some molecules participating in oligodendrocytes and the roles of OPC during homeostasis and dyshomeostasis are presented in Figure 2.

### Immunomodulatory Functions of Oligodendrocyte Lineage Cells

In contrast to microglia cells, but similar to astrocytes, oligodendrocytes and OPCs belonging to the same cell lineage are commonly classified as non-immune glial cells. However, recent discoveries shed new light on the immune properties of oligodendrocyte lineage cells including the regulation of innate and adaptive responses. Reciprocally, both adaptive and innate immune cells can shape the fate of OPCs via a range of immune mediators and trophic factors (104). Together with other glial cells, OPCs play a role in modulating the immune response by expression of immune-associated genes, response to immune stimuli, phagocytosis, antigen presentation, and production of immunomodulating factors (102, 105). In normal conditions, oligodendrocytes express Toll-like receptors (TLRs) that might allow for participation in the innate responses (106). So far, TLR2 and TLR3 create a unique composition of TLRs expressed by oligodendrocytes, and their abnormal activation may directly or indirectly affect other brain cells leading to different brain pathologies such as AD (107).

Oligodendrocytes are the most susceptible cells of the CNS, and mainly due to oxidative damage and their own high metabolic speed and vast oxygen usage, they needed to produce a bulk of lipids (108). Additionally, oligodendrocyte injuries and death can be triggered by actions of microglia and astrocytes. Nevertheless, they can actively protect themselves from harsh environments, but most importantly, they can modulate and shape immune responses and function of other brain cells. It has been found that the key transcription factor regulating the main functions of oligodendrocytes, improving their survival and maturation, is nuclear factor-kB (NF-kB). Since NF-kB is a master regulator of a variety of genes including immune-related ones, it is postulated that oligodendrocytes directly or indirectly influence brain immunity (109). It is worth mentioning that NF-kB can be activated not only by inflammatory cytokines such as TNF-α but also by neurotransmitters and neurotrophic factors such as nerve growth factor (NGF) (110). Brain-derived growth factor (BDNF) and leukemia inhibitory factor (LIF) increase OPC differentiation and boost myelin repair (111, 112). In this context, oligodendrocyte lineage cells are also responsive to factors produced by regulatory T and B cells that improve remyelination and repair processes (113, 114). Still quite controversial is the contribution of NF-kB in the myelination process in the CNS. Several studies point out the indirect contribution of the oligodendrocytic NF-kB pathway to normal and pathologic myelination, in contrast to astrocytic and microglial NF-kB, which directly boosts demyelination (109). A wide range of factors involved in immune responses, including various cytokines, chemokines, complement, antigen-presenting molecules, tetraspanins, as well as neuroimmune regulatory proteins, have been shown to be expressed by oligodendrocytes (115). The ability of oligodendrocytes to produce IL-17 can influence autoimmunity since dysregulation of IL-17 has been linked to development of MS (116). A myriad of other proinflammatory cytokines such as IL-1β, IL-18, and IL-6; chemokine receptors; chemokines such as CCL2, CCL3, and CXCL12; and others produced by oligodendrocyte lineage cells under specified conditions for immune cell migration advocate for their potential role as critical players in innate and acquired immunity (104).

## An Innate Arm of Immunity in Brain Disease

Glial cells as part of innate immunity are crucial players in a wide array of neurological disorders. Their primarily role in the brain is to protect the CNS from any insult and repair the nervous tissue after the injury.

### Astrogliosis, a Double-Edged Sword

Astrocytes were found to respond to stimuli by changing morphology, upregulation of MHC and GFAP, and immune mediators' release. Astrocytes are in close contact with blood vessels and control and guard the infiltration of cells from the blood to CNS parenchyma (117). Astrocytes can serve as APCs and upregulate MHC I and II (118). It has been found that oxidative stress is the main force for astrocytic activation (119). The term astrogliosis refers to a physiological defense response of astrocytes to neurological disorders (120). This process aims for the inhibition of the spread of inflammatory cells, repair of BBB, and scar formation. During astrogliosis, the formation of new neurons and oligodendrocytes from stem-like reactive astrocytes has been observed (121). Additionally, the scar formation may stimulate axonal regrowth after severe spinal cord injury (122). During MS progression, astrocytes produce and release anti-inflammatory molecules like IL-10 and TGF-β leading to resolution of the inflammatory responses (123). Although glial scar formation promotes axonal regeneration after spinal cord injury in mice (122), the study using the murine model for MS shows that remyelination occurs despite an abundant glial reaction (124). The exact effect of reactive astrogliosis in disease is complex and can range from beneficial to harmful. During acute stress or focal cerebral ischemia, this process is beneficial. However, it may have unfavorable outcome during regeneration. Excessive astrogliosis may be neurotoxic by producing reactive oxygen species or proinflammatory cytokines. Elimination of activated astrocytes improved axonal regeneration after injury in mice (125). Atrophic astrocytes with loss of function are hallmarks for many chronic neurological disorders. Deciphering the precise role of activated astrocytes in different neurological diseases is currently difficult due to the lack of specific markers for highly heterogeneous, region-specific astrocyte subtypes.

### Microglia in Neurological Disorders

Microglia and non-parenchymal macrophages, after stimulation, change their morphology and resemble systemic macrophages, upregulate MHCs, and release cytokines and nitric oxide. Recent exciting discoveries based on new genetic, molecular, and pharmacological tools allow for a better understanding of microglia phenotypes during an injury or disease that can be detrimental or beneficial (126). Infection-related damage efficiently triggers microglia activation with inflammasome activation, a mechanism that keeps control of infection in the CNS. Several studies show severe disease progression in animal models with microglia deletion (127, 128). AD is the most frequent neurodegenerative disorder and the leading cause of dementia. Characteristic features of AD are parenchymal depositions of Aβ aggregates in the form of plaques and aggregates of tau protein, creating intraneural neurofibrillary tangles. Inflammation in neurodegenerative disorders like AD primarily relates to the innate immune system compared to classic inflammatory diseases such as MS (129). In neurodegenerative diseases, large-scale genome-wide association studies (GWAS) show more than 20 loci in immune-related genes mostly expressed in microglia or myeloid cells (15). These genes also create a pool of risk factors for neurodegenerative diseases. One of the most pronounced risk factors for neurodegenerative diseases is mutated TREM2. TREM2 is an innate immune receptor expressed by myeloid cells, including microglia. During the early stages of brain development, TREM2 has been found to play a key role in eliminating extra synapses by regulation of microglial activity, adding to synaptic remodeling and plasticity described above (35). TREM2 signaling suppresses inflammatory responses in microglia by reducing cytokine production and increasing phagocytic activity that might lead to a reduction in Aβ deposition and limitation of neurodegeneration. Using optical imaging studies, microglia have been found to create a protective barrier at an early stage of the disease (130). Studies on mice and human subjects with R47H TREM2 mutations confirmed that microglia surround amyloid plaques and consequently limit plaque-associated neuritic dystrophy (131). Of note, the R47H variant of TREM2 is one of the strongest single allele genetic risk factors for AD, Parkinson's disease, amyotrophic lateral sclerosis (ALS), and frontotemporal dementia (FTD). Recently, Jay et al. (132) demonstrated that TREM2 deficiency in regard to Aβ deposition plays a different role depending on the stage of disease in the mouse model of AD. It seems that amyloid plaques in the early stage of the disease activate microglia, which protect against disease; however, in the long run, plaques accumulate and stimulate the inflammasome in microglia, causing malfunctions and disease progression. The NLRP3 inflammasome is fundamental for IL-1β maturation and inflammatory responses caused by β-amyloid (Aβ) depositions in AD. In APP/PS1AD transgenic mice, deletion of caspase-1 or NLRP3 reduced amyloid depositions (133). An important role for TREM2 is to sustain metabolic fitness, energy homeostasis, proliferation, and survival in microglia through mTOR signaling. Ulland et al. showed that TREM2 deficiency in 5XFAD mouse model of AD causes metabolic and energetic imbalance followed by increased autophagy that results in a stressed and dysfunctional microglia state (134). Wendeln et al. (135), for the first time, showed that immune memory occurs in the mouse brain via epigenetic changes and that it is predominantly mediated by microglia with an impact on neuropathology. This epigenetic reprogramming followed by inflammatory stimuli may induce immune training or tolerance and change microglial responses to β-amyloid depositions. Training promotes while tolerance alleviates neuropathology.

Gliosis is one of the characteristics of MS, and involvement of innate immunity is evident. Microglia respond to tissue damage by MHC class I, II, and co-stimulatory molecule upregulation, the release of cytokines and chemokines that further attract a plethora of cells like T cells, B cells, monocytes, and dendritic (DC)-like cells that add to the myelin sheath destruction by nitric oxide, and matrix metalloproteinases. In EAE, microglia react to injury through increased proliferation, directed migration, phagocytosis, activation of the NLRP3 inflammasome, and, consequently, the release of proinflammatory mediators (136). Both tissue-resident and recruited macrophages play a key role in EAE as their depletion leads to diminution of the disease (137). Moreover, depletion of monocyte-derived cells in EAE has a therapeutic effect on long-term axonal loss (138). However, it has been shown that antigen presentation by microglia is not crucial for disease induction as shown by experiments with mice lacking MHCII in microglia, but not peripheral myeloid cells (139). So far, microglia's involvement vs. recruited myeloid cells in the activation of CD4+T cells has not been resolved. It has been proven that in the animal model driven by CD8+ T cells, the microglial involvement is crucial (140). Recent studies using advanced technologies such as single-cell RNA sequencing and mass cytometry show that in EAE and MS, microglia and macrophages display complex heterogeneity that may lead to both immune-mediated inflammation and regeneration (141). In active MS lesions, the correlation between activated microglia and macrophages, demyelination, and axonal loss has been observed. However, active tissue injury also coincided with upregulation of anti-inflammatory and tissue-repair processes (142).

### Oligodendrocyte Lineage Cells in a Disease Context

Activated by immune stimuli, similar to astrocytes and microglia, OPCs undergo morphological and phenotypic changes such as shortening of processes, adopting ameboid shape, and expression of CD11b (143). During adult cerebral cortex injury, OPCs differentiate preferentially into astrocytes and participate in glial scar formation (144). After insult, NG2^+^ cells contribute to re-myelination (145). In MS and in a lysolecithin model of demyelination, aberrant OPC perivascular migration was detected, leading to disruption of BBB and CNS inflammation (146). NG2^+^ glial cells respond to systemic inflammation after LPS treatment and exposure to proinflammatory cytokines like IL-1β and IL-6, by reduced proliferation (147).

Recently, a single-cell transcriptomic analysis of oligodendrocyte lineage cells from the spinal cord of mice with EAE revealed unique OPCs that expressed several genes associated with immune cells such as genes involved in antigen processing and presentation, and genes involved in immunoprotection (105). These distinct, disease-associated OPCs found also in human MS brains acquired phagocytic abilities and, by expressing MHC-II, possess the ability to boost primary and memory CD4^+^ T cells and express known MS susceptibility genes. Of note, many genes detected in oligodendrocyte lineage cells were those belonging to MS susceptibility genes (148). In proinflammatory disease settings, OPCs acquire the ability to present antigen in the context of both MHC class I and class-II (102). Human studies in several neurological disorders based on single-cell and single-nucleus RNA sequencing revealed proinflammatory characteristics of OPCs. However, a recent single-nucleus RNA-sequencing study done on frozen tissue from healthy individuals and MS patients revealed several different, highly heterogeneous mature oligodendrocyte sub-clusters with upregulated immune genes in both diseased and normal tissue (149). How the existence of such heterogeneous functional states of oligodendrocytes contributes to health and disease is still a conundrum. Another recent study of MS lesions using the same technique showed a signature of stressed oligodendrocytes and damages to neurons in upper-cortical layers underlying meningeal inflammation (150).

The process of demyelination and consequent remyelination with different intensities dependent on disease phase and extent of damage are key features of MS. There is an ongoing debate on whether the remyelination process is dependent on recruitment of OPCs or on surviving, existing oligodendrocytes. Recent imaging and histologic studies on human samples show a quite limited process of OPC migration, differentiation to oligodendrocytes and ensheathing injured axons (104). OPCs have been found to gather around acute lesions; however, in chronic MS lesions, their number is limited (151). Such “differentiation block” and reduced numbers of OPC's in chronic MS lesions leading to remyelination failure can be a consequence of their high vulnerability to metabolic stress as mentioned above, or/and an intrinsic and systemic compartment attack (152, 153). Cytotoxic factors produced by MS B cells have been found to be detrimental to OPCs differentiation and survival (154). Accumulating evidence suggests, however, that naturally occurring IgM in mice and humans possesses remyelination-promoting abilities (155). Direct and/or indirect involvement of astrocytes and microglia can also be responsible for OPC death. Reactive astrocytes induced by activated microglia may kill OPCs and block their differentiation (156). One of the cytokines that allow cross-talk between OPCs and astrocytes is IL-17, which, via Notch signaling, enhances proliferation and inflammatory gene expression and impairs OPCs differentiation (157). A similar effect on OPC differentiation is attributed to IFN-γ. The role of IFN-γ as an inducer of immune transition of oligodendrocyte lineage cells is still controversial since its effect on oligodendrocyte lineage cells is mostly related to upregulation of MHC molecules and inhibition of differentiation (158). IFN-γ in a cuprizone-induced demyelination mouse model in EAE prevented re-myelinization (159) and astrocytic TNF-α-induced apoptosis (160). One of the crucial factors regulating proinflammatory properties and influencing the myelination process is low-density lipoprotein receptor-related protein (LRP1). LRP1 knock-out mice in EAE and the cuprizone model display lower inflammation and CD8+ cell proliferation and increased myelination (161). Oligodendrocyte lineage cells are not the only crucial players during MS development and progression but they also may perform an important role in neurodegenerative disorders. In a mouse model of AD, a new subpopulation of serpina3+/C4b+ reactive oligodendrocytes has been detected (162). Additionally, oligodendrocytes in AD have been found to express granulocyte activation genes (163). In a mouse model of Parkinson's disease, deficiency of NG2^+^ cells resulted in increased neuroinflammation and nigral dopaminergic neuron loss (103). These immunomodulatory properties of oligodendrocyte lineage cells are exceptionally important in the context of neuroinflammation and neurodegenerative disorders and may open new opportunities for research and therapeutic modalities.

Unfortunately, glia research in its current form using rodent models and *in vitro* settings poorly mimics human neuropathology (70, 71). The vast majority of animal models are based on young animals, mostly with aggressive phenotypes of disease, which, in the case of AD, an age-related neurodegenerative disease, is not suitable. Recently, the gene expression profiling data obtained from post-mortem human AD specimens of superior frontal gyrus demonstrated a lack of resemblance to disease activation-related gene profiles from animal models except genes involved in lipid/lysosomal function (164). In contrast, this profile of human genes called Alzheimer's microglia/myeloid cells (HAM) significantly resembled an “enhanced human aging” phenotype. The oxidative stress that damages mitochondria is postulated to be the main mediator of an energy crisis and upregulation of stress-response genes observed in cognitive decline in neurodegenerative disorders (165).

## An Adaptive Arm of Immunity in Brain Health

**Figure 3:**
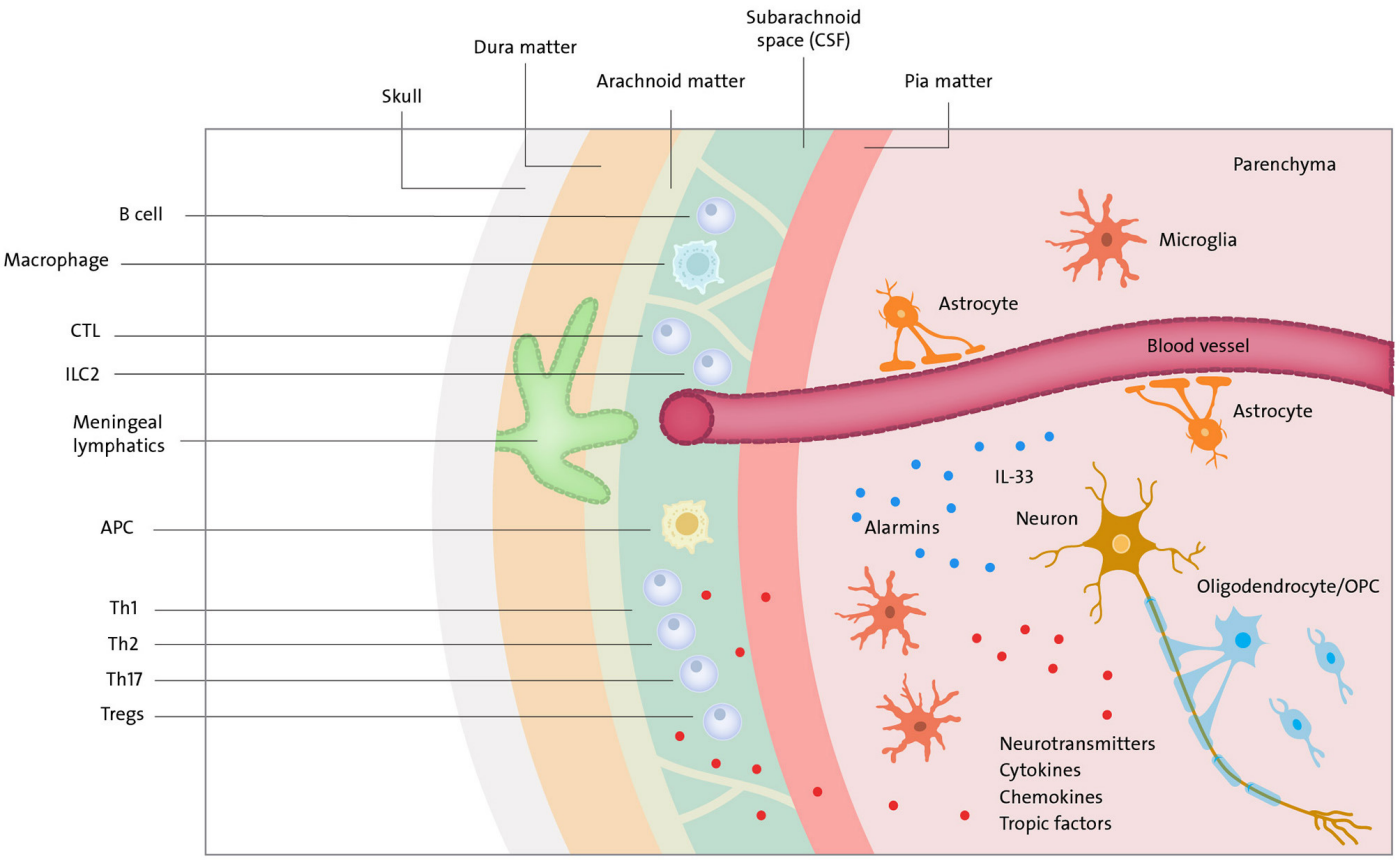
Neuroimmune cross-talk. Meningeal lymphatics and blood vessels (in the brain parenchyma covered by BBB) drain macromolecules and immune cells (T cells, B cells, ILC2, APC, etc.) to CSF present in subarachnoid space. Immune compounds can further travel to lymph nodes of head & neck where they encounter the rest of the immune system. Peripheral immune cells and cells in brain parenchyma can communicate via cytokines, chemokines, tropic factors and neurotransmitters. T cells via norepinephrine—choline acetyltransferase T cell-acetylcholine axis can suppress immune response. Neurons can communicate a danger by release of alarmins (e.g., IL-33).

The discovery of the functional lymphatic system in the mouse brain's meninges changed our perception of cross-talk between CNS and the adaptive immune system (10, 11). The brain is separated from the peripheral immune system by cerebrospinal fluid (CSF) and meninges. The latter is composed of the dura, the arachnoid, and the pia mater. CSF is present in the subarachnoid space between the arachnoid and pia mater. CSF and meningeal borders are rich in peripheral lymphocytes such as T cells, CD8+ cells, CD4+ cells, natural killer (NK) cells, B cells, and natural killer T (NKT) cells (166) (Figure 3). Although the precise ways for immune cells and macromolecules to leave and enter the CNS are still under discussion, a recent study on EAE shows that meningeal lymphatics allow the drainage of cerebrospinal fluid components and enable immune cells to enter draining lymph nodes in a CCR7-dependent manner (167). At the same time, CSF and interstitial fluid (ISF) directly connect the CNS with the head and neck lymph nodes carrying main components of innate as well as adaptive immune cells like T cells, including regulatory CD25+ FoxP3+ cells, B cells, macrophages, and dendritic cells (168). In normal conditions, these immune cells assure proper surveillance to maintain brain function and homeostasis.

### T Cells and Their Cytokines Regulate Neuronal Function

T cells through cytokine signaling clear viruses from the CNS and regulate brain functions such as spatial learning. Despite the importance of microglia in CNS homeostasis, the removal of microglia at baseline does not change cognition and behavioral tasks in adult mice (169). On the contrary, it has been found that the removal of adaptive immunity can harm cognitive functions. The main role in this process is postulated for CD4+T cells and the cytokines that they secrete in meningeal spaces, which modulate immune responses and control neuronal activity, including learning and social behavior (168, 170). Studies using B-cell-deficient mice demonstrated a minimal role of B cells in supporting learning behaviors (171). The importance of adaptive immunity in cognitive function has been demonstrated using experiments with passive transfers of T cells (172–174). A critical role has been assigned to T-cell-derived IL-4 in regulating cognitive function by influencing meningeal myeloid cell phenotype (175). Lack of T cells from meningeal spaces or T cells' inability to produce IL-4 was related to proinflammatory phenotype by meningeal myeloid cells and cognitive impairment. The ability of T cells to infiltrate the meninges is a key event for optimal T cells influence on cognition as the blockage of their entry impairs this function (175). Recently, the role of CD4+ T-cell-derived IL-13 cytokine in learning and memory, by acting on astrocytes to produce BDNF, which fosters cognitive functions, has been documented (176). Moreover, different *in vitro* and *in vivo* approaches show that certain other anti-inflammatory cytokines, such as IL-10 and IL-4, possess a neuroprotective effect, inducing neurogenesis and synapse remodeling (177). Interestingly, the autoimmune nature of pro-cognitive T cells has been proposed because only MOG-reactive T cells within meningeal barriers and not ovalbumin-specific T cells mediated the beneficial effect (171). This is in line with an initial study where autoimmune T cells specific to myelin basic protein were neuroprotective and could protect injured neurons in rats (178). Another study using murine model and culture-based systems demonstrated that T cell-derived IL-4 in an antigen-independent way protects and induces recovery of axonal damage by activating neuronal IL-4 receptors, which potentiated neurotrophin signaling (179). It is important to note that environmental factors like prenatal gut microbiota might influence offspring murine brain development via cytokines released by T cells (180). Maternal inflammation with T cells producing IL-17 lead to behavioral abnormalities (181). The negative implication of social behavior is documented for IL-17 during brain development in mice (181). IL-17 production can be driven by several different cytokines, such as IL-6, IL-21, IL-23, and TGF-β (182). Studies show the influence of microbiota on microglia maturation and function as well as autoimmune mechanisms in the CNS (183).

### Role of IFNs in Brain Homeostasis

As presented above, T cells and some Th2 cytokines possess neuroprotective functions and control learning and memory tasks, whereas the control of social behavior is attributed more to T-cell-derived IFN-γ. IFN-γ knockout mice display aberrant social behavior as this cytokine stimulated inhibitory neurons and increased GABAergic currents in projection neurons. This finding can have an important implication for autism spectrum disorders with aberrant neuronal connectivity and social dysfunction (184). On the other hand, in the old mouse brain, decline in function might be related to IFN-γ secreted by T cells inhibiting neural stem cells within neurogenic niches (185). Moreover, studies with neurotropic flaviviruses demonstrated post-infectious cognitive sequelae leading to spatial-learning defects in animals driven by CD8+T cells or T-cell-derived IFN-γ (186). Members of other type interferons such as the subfamily members of IFN-α and IFN-β originating from different sources including lymphocytes are vital in maintaining brain homeostasis. In human and mouse neuronal culture, IFN-β, a type I IFN, inhibited BDNF and, thus, the neurite outgrowth and survival (187). The role of IFN type I family in the CNS is quite enigmatic since correct levels of every member of the family are needed to maintain tissue functionality. Nevertheless, their chronic release and permanent microglia stimulation have been linked to the development of distinct human neurological diseases, termed “cerebral cytokinopathies” (188). Interestingly, the abundance of IFN-α subfamily members or their administration changes circadian rhythm, the opioid receptor system, and thermoregulation, whereas the IFNs' absence has no effect on these functions (189).

It is increasingly clear that the adaptive immunity and cytokines derived from T cells and other sources control the development and function of the CNS (190). Recently, the role of ILC2 (type 2 innate lymphoid cells) within the meninges has been discussed (191). Alarmins together with neuropeptide NMU released by neurons can expand ILC2, the only immune cells expressing the NMU receptor (192). Many questions still exist about the homeostatic or inflammatory boosting abilities of ILC2 cells.

## An Adaptive Arm of Immunity in Brain Disease

The majority of our understanding of adaptive immunity in CNS malfunction comes from the prototypic inflammatory disease—MS and its animal model (experimental autoimmune encephalomyelitis) EAE (193–198).

### Adaptive Immune Sentinels in MS and EAE

MS is an autoimmune disorder of the CNS caused by an aberrant, chronic progressive inflammatory response leading to demyelination and permanent neurological disability in young adults, especially women. The pathogenesis of MS possesses a strong immune component supported by the efficacy of selective and non-selective immunosuppressive therapies on the disease progression. However, the innate or adaptive immune system's contribution to the development and progression of the disease has not yet been fully elucidated. Immunological, genetic, and histopathological studies in mice and humans suggested a dominant involvement of peripheral adaptive immunity during the early phase of disease and innate immune reactions within the CNS during the progressive stage (199). Although traditionally MS is portrayed as a T-cell-mediated disease over years of research, it becomes evident that a myriad of immune cells are responsible for MS pathology including B cells, CD8+ and CD4+ T cells, monocytes, and macrophages, as well as NK cells and neutrophils (200–204). One of the central differences between EAE and MS is that in EAE, T cell inflammation is dominated by CD4+T cells (205), whereas in MS, it is dominated by CD8+ T cells (206) and B cells (123). Recently, involvement of innate-like T lymphocytes, unconventional T cells with characteristics of innate and adaptive cells in MS pathology, has been suggested (207). Most likely initiated by autoreactive T cells, the immune response is facilitated by both peripheral and immune cells residing in the CNS. One of the crucial players linking adaptive with innate immunity is the inflammasome complex. NLRP3 inflammasome has been found to be critical for EAE induction as shown by studies with NLRP3 and IL-1 knockout mice most likely through effects on caspase-1-dependent cytokines, which then influence Th1 and Th17 cells (208). The protective mechanisms of current MS therapies such as IFN-β or glatiramer partially rely on dampening the IL-1β levels.

Besides obvious inflammatory mechanisms in MS, this disease also has characteristics of neurodegeneration. Earlier, it was believed that neurodegeneration is secondary to inflammatory-demyelinating processes, but now, it is widely accepted that axonal loss proceeds in parallel with induction of acute white matter plaques (209). Several mechanisms contribute to neurodegeneration in MS, like redistribution of ion channels, proinflammatory mediators, ROS causing mitochondrial dysfunction, and neuronal metabolic imbalance caused in part by glutamate produced by meningeal infiltrating immune cells (210).

### Protective Role of T Cells in Different Neurological Disorders

For a long time, it has been established that CD4+ T cells play a major role in preventing spontaneous experimental autoimmune encephalomyelitis (Sp-EAE) (211, 212) as well as spontaneous autoimmune disease such as non-obese diabetes in mice (213). Transgenic mice with T cell receptors specific for myelin basic protein (MBP)-Ac1-11 do not develop EAE unless actively immunized with myelin basic protein (MBP). However, when backcrossed to the RAG-1 background, these mice spontaneously develop an acute form of progressive EAE (211). Our previous study demonstrated that three different populations of splenocytes isolated from AV4/BV8S2 double transgenic mice immunized with MBP possess the ability to protect from spontaneous EAE (214). The most effective in regulating EAE was the population with a CD4+CD25+CCR4+ phenotype. An ultimate regulatory activity has been consistently demonstrated for Tregs in inflammatory conditions, including EAE and MS (215). Activation of endogenous Tregs and recovery from EAE were proven for B cells and mechanisms involving IL-10 (216). The first study on the role of meningeal drainage in the context of neuroinflammation in EAE shows the importance of this route for CNS drainage of antigens and immune cells as well as importantly, shaping the phenotype of T cells (167). The CNS fluid drainage of CSF antigens has been described elsewhere (217).

Accumulating evidence shows that in classic neurodegenerative diseases such as AD and ALS, besides phagocytes, a crucial role is played by the adaptive immune system, which contributes to disease progression and protection. Mice models of AD and ALS, deficient in T cells, display rapid disease progression (218, 219). Some early experiments in murine models point out the importance of adaptive immunity in nerve injury (178, 220). It has been shown that T cell response in CNS injury might be TCR-dependent as well as TCR-independent depending on experimental conditions (178, 179). In the CNS injury model, the adaptive response follows the activation of glia and the release of IL-33, a monocyte recruitment cytokine most abundantly present in the nervous system and mice lacking IL-33 have impaired recovery after CNS injury, which is the consequence of reduced myeloid cell infiltrates (221).

## Concluding Remarks

Rapid progress in high-resolution live image technologies, single-cell genomics, quantitative proteomics, and epigenetics brings discoveries in the field of neurology. The long-lasting dogma imposing separation of the peripheral immune system from the nervous system, linking these two only in extreme pathological conditions, becomes obsolete. Their co-existence and close interplay assure proper functioning and homeostasis of the CNS. Failure of clinical trials of CNS therapeutics (with the possible exception of MS), mostly immunosuppressive and anti-inflammatory, proves our poor understanding of immune sentinels' function in the CNS. It is time to reconsider the immune system as a close partner to the brain environment since it is crucial for brain development, homeostasis, and disease. Recent discoveries of meningeal lymphatic vessels changed our perception of cross-talk between CNS and the immune system. How meningeal immunity and reciprocal cross-talk between innate and adaptive immunity influence CNS health and disease is now of critical importance in neuroimmunology.

## Author Contributions

AM wrote the first and final drafts, performed data mining, created figures, and contributed to editing. All authors contributed to the article and approved the submitted version.

## Conflict of Interest

The authors declare that the research was conducted in the absence of any commercial or financial relationships that could be construed as a potential conflict of interest.

## References

[1] Cooper MD Alder MN. The evolution of adaptive immune systems. Cell. (2006) 124:815–22. 10.1016/j.cell.2006.02.001 16497590

[2] Hoftberger R Lassmann H. Immune-mediated disorders. Handb Clin Neurol. (2017) 145:285–99. 10.1016/B978-0-12-802395-2.00020-1 28987176

[3] Barichello T Generoso JS Simoes LR Goularte JA Petronilho F Saigal P. Role of microglial activation in the pathophysiology of bacterial meningitis. Mol Neurobiol. (2016) 53:1770–81. 10.1007/s12035-015-9107-4 25744564

[4] Hammond TR Marsh SE Stevens B. Immune signaling in neurodegeneration. Immunity. (2019) 50:955–74. 10.1016/j.immuni.2019.03.016 30995509 PMC6822103

[5] Sankowski R Mader S Valdes-Ferrer SI. Systemic inflammation and the brain: novel roles of genetic, molecular, and environmental cues as drivers of neurodegeneration. Front Cell Neurosci. (2015) 9:28. 10.3389/fncel.2015.00028 25698933 PMC4313590

[6] Kaplan HJ Niederkorn JY. Regional immunity and immune privilege. Chem Immunol Allergy. (2007) 92:11–26. 10.1159/000099237 17264479

[7] Medawar PB. Immunity to homologous grafted skin; the fate of skin homografts transplanted to the brain, to subcutaneous tissue, and to the anterior chamber of the eye. Br J Exp Pathol. (1948) 29:58–69. 18865105 PMC2073079

[8] Carson MJ Doose JM Melchior B Schmid CD Ploix CC. CNS immune privilege: hiding in plain sight. Immunol Rev. (2006) 213:48–65. 10.1111/j.1600-065X.2006.00441.x 16972896 PMC2633103

[9] Wekerle H. Breaking ignorance: the case of the brain. Curr Top Microbiol Immunol. (2006) 305:25–50. 10.1007/3-540-29714-6_2 16724799

[10] Aspelund A Antila S Proulx ST Karlsen TV Karaman S Detmar M. A dural lymphatic vascular system that drains brain interstitial fluid and macromolecules. J Exp Med. (2015) 212:991–9. 10.1084/jem.20142290 26077718 PMC4493418

[11] Louveau A Smirnov I Keyes TJ Eccles JD Rouhani SJ Peske JD. Structural and functional features of central nervous system lymphatic vessels. Nature. (2015) 523:337–41. 10.1038/nature14432 26030524 PMC4506234

[12] Negi N Das BK. CNS: Not an immunoprivilaged site anymore but a virtual secondary lymphoid organ. Int Rev Immunol. (2018) 37:57–68. 10.1080/08830185.2017.1357719 28961037

[13] Garay PA McAllister AK. Novel roles for immune molecules in neural development: implications for neurodevelopmental disorders. Front Synaptic Neurosci. (2010) 2:136. 10.3389/fnsyn.2010.00136 21423522 PMC3059681

[14] Crotti A Ransohoff RM. Microglial physiology and pathophysiology: insights from genome-wide transcriptional profiling. Immunity. (2016) 44:505–15. 10.1016/j.immuni.2016.02.013 26982357

[15] Ransohoff RM. How neuroinflammation contributes to neurodegeneration. Science. (2016) 353:777–83. 10.1126/science.aag2590 27540165

[16] Matejuk A Dwyer J Hopke C Vandenbark AA Offner H. Opposing roles for TGF-beta1 and TGF-beta3 isoforms in experimental autoimmune encephalomyelitis. Cytokine. (2004) 25:45–51. 10.1016/j.cyto.2003.09.007 14693159

[17] Sun L He C Nair L Yeung J Egwuagu CE. Interleukin 12 (IL-12) family cytokines: role in immune pathogenesis and treatment of CNS autoimmune disease. Cytokine. (2015) 75:249–55. 10.1016/j.cyto.2015.01.030 25796985 PMC4553122

[18] Burmeister AR Marriott I. The interleukin-10 family of cytokines and their role in the CNS. Front Cell Neurosci. (2018) 12:458. 10.3389/fncel.2018.00458 30542269 PMC6277801

[19] Scanzano A Cosentino M. Adrenergic regulation of innate immunity: a review. Front Pharmacol. (2015) 6:171. 10.3389/fphar.2015.00171 26321956 PMC4534859

[20] Delpech JC Herron S Botros MB Ikezu T. Neuroimmune crosstalk through extracellular vesicles in health and disease. Trends Neurosci. (2019) 42:361–72. 10.1016/j.tins.2019.02.007 30926143 PMC6486849

[21] Kanninen KM Bister N Koistinaho J Malm T. Exosomes as new diagnostic tools in CNS diseases. Biochim Biophys Acta. (2016) 1862:40310. 10.1016/j.bbadis.2015.09.020 26432482

[22] Deverman BE Patterson PH. Cytokines and CNS development. Neuron. (2009) 64:61–78. 10.1016/j.neuron.2009.09.002 19840550

[23] Bauer S Kerr BJ Patterson PH. The neuropoietic cytokine family in development, plasticity, disease and injury. Nat Rev Neurosci. (2007) 8:221–32. 10.1038/nrn2054 17311007

[24] Carpentier PA Palmer TD. Immune influence on adult neural stem cell regulation and function. Neuron. (2009) 64:79–92. 10.1016/j.neuron.2009.08.038 19840551 PMC2789107

[25] Ginhoux F Greter M Leboeuf M Nandi S See P Gokhan S. Fate mapping analysis reveals that adult microglia derive from primitive macrophages. Science. (2010) 330:841–5. 10.1126/science.1194637 20966214 PMC3719181

[26] Ransohoff RM Schafer D Vincent A Blachere NE Bar-Or A. Neuroinflammation: ways in which the immune system affects the brain. Neurotherapeutics. (2015) 12:896–909. 10.1007/s13311-015-0385-3 26306439 PMC4604183

[27] Wang Y Szretter KJ Vermi W Gilfillan S Rossini C Cella M. IL-34 is a tissue-restricted ligand of CSF1R required for the development of Langerhans cells and microglia. Nat Immunol. (2012) 13:753–60. 10.1038/ni.2360 22729249 PMC3941469

[28] Butovsky O Jedrychowski MP Moore CS Cialic R Lanser AJ Gabriely G. Identification of a unique TGF-beta-dependent molecular and functional signature in microglia. Nat Neurosci. (2014) 17:131–43. 10.1038/nn.3599 24316888 PMC4066672

[29] Buttgereit A Lelios I Yu X Vrohlings M Krakoski NR Gautier EL. Sall1 is a transcriptional regulator defining microglia identity and function. Nat Immunol. (2016) 17:1397–406. 10.1038/ni.3585 27776109

[30] Endo F Komine O Fujimori-Tonou N Katsuno M Jin S Watanabe S. Astrocyte-derived TGF-beta1 accelerates disease progression in ALS mice by interfering with the neuroprotective functions of microglia and T cells. Cell Rep. (2015) 11:592–604. 10.1016/j.celrep.2015.03.053 25892237

[31] Wlodarczyk A Holtman IR Krueger M Yogev N Bruttger J Khorooshi R. A novel microglial subset plays a key role in myelinogenesis in developing brain. EMBO J. (2017) 36:3292–308. 10.15252/embj.201696056 28963396 PMC5686552

[32] Hagemeyer N Hanft KM Akriditou MA Unger N Park ES Stanley ER. Microglia contribute to normal myelinogenesis and to oligodendrocyte progenitor maintenance during adulthood. Acta Neuropathol. (2017) 134:441–58. 10.1007/s00401-017-1747-1 28685323 PMC5951721

[33] Lloyd AF Davies CL Holloway RK Labrak Y Ireland G Carradori D. Central nervous system regeneration is driven by microglia necroptosis and repopulation. Nat Neurosci. (2019) 22:1046–52. 10.1038/s41593-019-0418-z 31182869 PMC6597360

[34] Salter MW Stevens B. Microglia emerge as central players in brain disease. Nat Med. (2017) 23:1018–27. 10.1038/nm.4397 28886007

[35] Filipello F Morini R Corradini I Zerbi V Canzi A Michalski B. The microglial innate immune receptor TREM2 is required for synapse elimination and normal brain connectivity. Immunity. (2018) 48:979–91.e978. 10.1016/j.immuni.2018.04.016 29752066

[36] Abiega O Beccari S Diaz-Aparicio I Nadjar A Laye S Leyrolle Q. Neuronal hyperactivity disturbs ATP microgradients, impairs microglial motility, and reduces phagocytic receptor expression triggering apoptosis/microglial phagocytosis uncoupling. PLoS Biol. (2016) 14:e1002466. 10.1371/journal.pbio.1002466 27228556 PMC4881984

[37] Stephan AH Barres BA Stevens B. The complement system: an unexpected role in synaptic pruning during development and disease. Annu Rev Neurosci. (2012) 35:369–89. 10.1146/annurev-neuro-061010-113810 22715882

[38] Hong S Beja-Glasser VF Nfonoyim BM Frouin A Li S Ramakrishnan S. Complement and microglia mediate early synapse loss in Alzheimer mouse models. Science. (2016) 352:712–6. 10.1126/science.aad8373 27033548 PMC5094372

[39] Vasek MJ Garber C Dorsey D Durrant DM Bollman B Soung A. A complement-microglial axis drives synapse loss during virus-induced memory impairment. Nature. (2016) 534:538–43. 10.1038/nature18283 27337340 PMC5452615

[40] Sekar A Bialas AR de Rivera H Davis A Hammond TR Kamitaki N. Schizophrenia risk from complex variation of complement component 4. Nature. (2016) 530:177–83. 10.1038/nature16549 26814963 PMC4752392

[41] Paolicelli RC Bolasco G Pagani F Maggi L Scianni M Panzanelli P. Synaptic pruning by microglia is necessary for normal brain development. Science. (2011) 333:1456–8. 10.1126/science.1202529 21778362

[42] Schafer DP Lehrman EK Kautzman AG Koyama R Mardinly AR Yamasaki R. Microglia sculpt postnatal neural circuits in an activity and complement-dependent manner. Neuron. (2012) 74:691–705. 10.1016/j.neuron.2012.03.026 22632727 PMC3528177

[43] Kopec AM Smith CJ Ayre NR Sweat SC Bilbo SD. Microglial dopamine receptor elimination defines sex-specific nucleus accumbens development and social behavior in adolescent rats. Nat Commun. (2018) 9:3769. 10.1038/s41467-018-06118-z 30254300 PMC6156594

[44] Chung WS Allen NJ Eroglu C. Astrocytes control synapse formation, function, and elimination. Cold Spring Harb Perspect Biol. (2015) 7:a020370. 10.1101/cshperspect.a020370 25663667 PMC4527946

[45] Stogsdill JA Ramirez J Liu D Kim YH Baldwin KT Enustun E. Astrocytic neuroligins control astrocyte morphogenesis and synaptogenesis. Nature. (2017) 551:192–7. 10.1038/nature24638 29120426 PMC5796651

[46] Allen NJ Eroglu C. Cell Biology of Astrocyte-Synapse Interactions. Neuron. (2017) 96:697–708. 10.1016/j.neuron.2017.09.056 29096081 PMC5687890

[47] Farhy-Tselnicker I Allen NJ. Astrocytes, neurons, synapses: a tripartite view on cortical circuit development. Neural Dev. (2018) 13:7. 10.1186/s13064-018-0104-y 29712572 PMC5928581

[48] Porter JT McCarthy KD. Astrocytic neurotransmitter receptors *in situ* and *in vivo*. Prog Neurobiol. (1997) 51:439–55. 10.1016/s0301-0082(96)00068-8 9106901

[49] Sun W McConnell E Pare JF Xu Q Chen M Peng W. Glutamate-dependent neuroglial calcium signaling differs between young and adult brain. Science. (2013) 339:197–200. 10.1126/science.1226740 23307741 PMC3569008

[50] Martin R Bajo-Graneras R Moratalla R Perea G Araque A. Circuit-specific signaling in astrocyte-neuron networks in basal ganglia pathways. Science. (2015) 349:730–4. 10.1126/science.aaa7945 26273054

[51] van Deijk AF Camargo N Timmerman J Heistek T Brouwers JF Mogavero F. Astrocyte lipid metabolism is critical for synapse development and function *in vivo*. Glia. (2017) 65:670–82. 10.1002/glia.23120 28168742

[52] Chung WS Clarke LE Wang GX Stafford BK Sher A Chakraborty C. Astrocytes mediate synapse elimination through MEGF10 and MERTK pathways. Nature. (2013) 504:394–400. 10.1038/nature12776 24270812 PMC3969024

[53] Bialas AR Stevens B. TGF-beta signaling regulates neuronal C1q expression and developmental synaptic refinement. Nat Neurosci. (2013) 16:1773–1782. 10.1038/nn.3560 24162655 PMC3973738

[54] Vainchtein ID Chin G Cho FS Kelley KW Miller JG Chien EC. Astrocyte-derived interleukin-33 promotes microglial synapse engulfment and neural circuit development. Science. (2018) 359:1269–73. 10.1126/science.aal3589 29420261 PMC6070131

[55] Stevens B Allen NJ Vazquez LE Howell GR Christopherson KS Nouri N. The classical complement cascade mediates CNS synapse elimination. Cell. (2007) 131:1164–78. 10.1016/j.cell.2007.10.036 18083105

[56] Matejuk A Leng Q Begum MD Woodle MC Scaria P Chou ST. Peptide-based antifungal therapies against emerging infections. Drugs Future. (2010) 35:197. 10.1358/dof.2010.035.03.1452077 20495663 PMC2873032

[57] Williams WM Castellani RJ Weinberg A Perry G Smith MA. Do beta-defensins and other antimicrobial peptides play a role in neuroimmune function and neurodegeneration? ScientificWorldJournal. (2012) 2012:905785. 10.1100/2012/905785 22606066 PMC3346844

[58] Matejuk A Ransohoff RM. Crosstalk between astrocytes and microglia: an overview. Front Immunol. (2020) 11:1416. 10.3389/fimmu.2020.01416 32765501 PMC7378357

[59] Goldmann T Wieghofer P Jordao MJ Prutek F Hagemeyer N Frenzel K. Origin, fate and dynamics of macrophages at central nervous system interfaces. Nat Immunol. (2016) 17:797–805. 10.1038/ni.3423 27135602 PMC4968048

[60] Prinz M Erny D Hagemeyer N. Ontogeny and homeostasis of CNS myeloid cells. Nat Immunol. (2017) 18:385–92. 10.1038/ni.3703 28323268

[61] Mildner A Schmidt H Nitsche M Merkler D Hanisch UK Mack M. Microglia in the adult brain arise from Ly-6ChiCCR2+ monocytes only under defined host conditions. Nat Neurosci. (2007) 10:1544–53. 10.1038/nn2015 18026096

[62] Bennett ML Bennett FC Liddelow SA Ajami B Zamanian JL Fernhoff NB. New tools for studying microglia in the mouse and human CNS. Proc Natl Acad Sci USA. (2016) 113:E1738–46. 10.1073/pnas.1525528113 26884166 PMC4812770

[63] Wong K Noubade R Manzanillo P Ota N Foreman O Hackney JA. Mice deficient in NRROS show abnormal microglial development and neurological disorders. Nat Immunol. (2017) 18:633–41. 10.1038/ni.3743 28459434

[64] Amit I Winter DR Jung S. The role of the local environment and epigenetics in shaping macrophage identity and their effect on tissue homeostasis. Nat Immunol. (2016) 17:18–25. 10.1038/ni.3325 26681458

[65] Bennett FC Bennett ML Yaqoob F Mulinyawe SB Grant GA Hayden Gephart M. A combination of ontogeny and CNS environment establishes microglial identity. Neuron. (2018) 98:1170–83.e1178. 10.1016/j.neuron.2018.05.014 29861285 PMC6023731

[66] Zhan L Krabbe G Du F Jones I Reichert MC Telpoukhovskaia M. Proximal recolonization by self-renewing microglia re-establishes microglial homeostasis in the adult mouse brain. PLoS Biol. (2019) 17:e3000134. 10.1371/journal.pbio.3000134 30735499 PMC6383943

[67] Hammond TR Dufort C Dissing-Olesen L Giera S Young A Wysoker A. Single-cell RNA sequencing of microglia throughout the mouse lifespan and in the injured brain reveals complex cell-state changes. Immunity. (2019) 50:253–71.e256. 10.1016/j.immuni.2018.11.004 30471926 PMC6655561

[68] Wimmer I Zrzavy T Lassmann H. Neuroinflammatory responses in experimental and human stroke lesions. J Neuroimmunol. (2018) 323:10–8. 10.1016/j.jneuroim.2018.07.003 30196821

[69] Masuda T Sankowski R Staszewski O Bottcher C Amann L Scheiwe C. Spatial and temporal heterogeneity of mouse and human microglia at single-cell resolution. Nature. (2019) 566:388–92. 10.1038/s41586-019-0924-x 30760929

[70] Ransohoff RM. All (animal) models (of neurodegeneration) are wrong. Are they also useful? J Exp Med. (2018) 215:2955–8. 10.1084/jem.20182042 30459159 PMC6279414

[71] Ransohoff RM. A polarizing question: do M1 and M2 microglia exist? Nat Neurosci. (2016) 19:987–91. 10.1038/nn.4338 27459405

[72] Nimmerjahn A Kirchhoff F Helmchen F. Resting microglial cells are highly dynamic surveillants of brain parenchyma *in vivo*. Science. (2005) 308:1314–8. 10.1126/science.1110647 15831717

[73] Li Y Du XF Liu CS Wen ZL Du JL. Reciprocal regulation between resting microglial dynamics and neuronal activity *in vivo*. Dev Cell. (2012) 23:1189–202. 10.1016/j.devcel.2012.10.027 23201120

[74] Izquierdo P Attwell D Madry C. Ion channels and receptors as determinants of microglial function. Trends Neurosci. (2019) 42:278–92. 10.1016/j.tins.2018.12.007 30678990

[75] Madry C Kyrargyri V Arancibia-Carcamo IL Jolivet R Kohsaka S Bryan RM. Microglial ramification, surveillance, and interleukin-1beta release are regulated by the two-pore domain K(+) channel THIK-1. Neuron. (2018) 97:299–312.e296. 10.1016/j.neuron.2017.12.002 29290552 PMC5783715

[76] Gu N Eyo UB Murugan M Peng J Matta S Dong H. Microglial P2Y12 receptors regulate microglial activation and surveillance during neuropathic pain. Brain Behav Immun. (2016) 55:82–92. 10.1016/j.bbi.2015.11.007 26576724 PMC4864135

[77] Hickman SE Kingery ND Ohsumi TK Borowsky ML Wang LC Means TK. The microglial sensome revealed by direct RNA sequencing. Nat Neurosci. (2013) 16:1896–1905. 10.1038/nn.3554 24162652 PMC3840123

[78] Ransohoff RM El Khoury J. Microglia in health and disease. Cold Spring Harb Perspect Biol. (2015) 8:a020560. 10.1101/cshperspect.a020560 26354893 PMC4691795

[79] Ewers M Franzmeier N Suarez-Calvet M Morenas-Rodriguez E Caballero MAA Kleinberger G. Increased soluble TREM2 in cerebrospinal fluid is associated with reduced cognitive and clinical decline in Alzheimer's disease. Sci Transl Med. (2019) 11:aav6221: 10.1126/scitranslmed.aav6221 PMC7050285 31462511

[80] Katsumoto A Lu H Miranda AS Ransohoff RM. Ontogeny and functions of central nervous system macrophages. J Immunol. (2014) 193:2615–21. 10.4049/jimmunol.1400716 25193935 PMC4157312

[81] Oberheim NA Takano T Han X He W Lin JH Wang F. Uniquely hominid features of adult human astrocytes. J Neurosci. (2009) 29:3276–87. 10.1523/JNEUROSCI.4707-08.2009 19279265 PMC2819812

[82] Kelley KW Nakao-Inoue H Molofsky AV Oldham MC. Variation among intact tissue samples reveals the core transcriptional features of human CNS cell classes. Nat Neurosci. (2018) 21:1171–84. 10.1038/s41593-018-0216-z 30154505 PMC6192711

[83] Zhang Y Sloan SA Clarke LE Caneda C Plaza CA Blumenthal PD. Purification and characterization of progenitor and mature human astrocytes reveals transcriptional and functional differences with mouse. Neuron. (2016) 89:37–53. 10.1016/j.neuron.2015.11.013 26687838 PMC4707064

[84] Oberheim NA Goldman SA Nedergaard M. Heterogeneity of astrocytic form and function. Methods Mol Biol. (2012) 814:23–45. 10.1007/978-1-61779-452-0_3 22144298 PMC3506190

[85] John Lin CC Yu K Hatcher A Huang TW Lee HK Carlson J. Identification of diverse astrocyte populations and their malignant analogs. Nat Neurosci. (2017) 20:396–405. 10.1038/nn.4493 28166219 PMC5824716

[86] Witcher MR Park YD Lee MR Sharma S Harris KM Kirov SA. Three-dimensional relationships between perisynaptic astroglia and human hippocampal synapses. Glia. (2010) 58:572–87. 10.1002/glia.20946 19908288 PMC2845925

[87] Foo LC Allen NJ Bushong EA Ventura PB Chung WS Zhou L. Development of a method for the purification and culture of rodent astrocytes. Neuron. (2011) 71:799–811. 10.1016/j.neuron.2011.07.022 21903074 PMC3172573

[88] Allen NJ Lyons DA. Glia as architects of central nervous system formation and function. Science. (2018) 362:181–5. 10.1126/science.aat0473 30309945 PMC6292669

[89] Araque A Parpura V Sanzgiri RP Haydon PG. Tripartite synapses: glia, the unacknowledged partner. Trends Neurosci. (1999) 22:208–215. 10.1016/s0166-2236(98)01349-6 10322493

[90] Khakh BS Sofroniew MV. Diversity of astrocyte functions and phenotypes in neural circuits. Nat Neurosci. (2015) 18:942–52. 10.1038/nn.4043 26108722 PMC5258184

[91] Masgrau R Guaza C Ransohoff RM Galea E. Should we stop saying 'glia' and 'neuroinflammation'? Trends Mol Med. (2017) 23:486–500. 10.1016/j.molmed.2017.04.005 28499701

[92] Stellwagen D Malenka RC. Synaptic scaling mediated by glial TNF-alpha. Nature. (2006) 440:1054–9. 10.1038/nature04671 16547515

[93] De Pitta M Brunel N Volterra A. Astrocytes: Orchestrating synaptic plasticity? Neuroscience. (2016) 323:43–61. 10.1016/j.neuroscience.2015.04.001 25862587

[94] Akay LA Effenberger AH Tsai LH. Cell of all trades: oligodendrocyte precursor cells in synaptic, vascular, immune function. Genes Dev. (2021) 35:180–98. 10.1101/gad.344218.120 33526585 PMC7849363

[95] Palazuelos J Klingener M Aguirre A. TGFbeta signaling regulates the timing of CNS myelination by modulating oligodendrocyte progenitor cell cycle exit through SMAD3/4/FoxO1/Sp1. J Neurosci. (2014) 34:7917–30. 10.1523/JNEUROSCI.0363-14.2014 24899714 PMC4044250

[96] Seo JH Maki T Maeda M Miyamoto N Liang AC Hayakawa K. Oligodendrocyte precursor cells support blood-brain barrier integrity via TGF-beta signaling. PLoS ONE. (2014) 9:e103174. 10.1371/journal.pone.0103174 25078775 PMC4117639

[97] Maheshwari A Janssens K Bogie J Van Den Haute C Struys T Lambrichts I. Local overexpression of interleukin-11 in the central nervous system limits demyelination and enhances remyelination. Mediators Inflamm. (2013) 2013:685317. 10.1155/2013/685317 23818742 PMC3683504

[98] Ding X Cao F Cui L Ciric B Zhang GX Rostami A. IL-9 signaling affects central nervous system resident cells during inflammatory stimuli. Exp Mol Pathol. (2015) 99:570–4. 10.1016/j.yexmp.2015.07.010 26216406 PMC6524950

[99] Rodgers JM Robinson AP Rosler ES Lariosa-Willingham K Persons RE Dugas JC. IL-17A activates ERK1/2 and enhances differentiation of oligodendrocyte progenitor cells. Glia. (2015) 63:768–79. 10.1002/glia.22783 25557204 PMC4400118

[100] Nishiyama A Komitova M Suzuki R Zhu X. Polydendrocytes (NG2 cells): multifunctional cells with lineage plasticity. Nat Rev Neurosci. (2009) 10:9–22. 10.1038/nrn2495 19096367

[101] Domingues HS Portugal CC Socodato R Relvas JB. Oligodendrocyte, astrocyte, and microglia crosstalk in myelin development, damage, and repair. Front Cell Dev Biol. (2016) 4:71. 10.3389/fcell.2016.00071 27551677 PMC4923166

[102] Kirby L Jin J Cardona JG Smith MD Martin KA Wang J. Oligodendrocyte precursor cells present antigen and are cytotoxic targets in inflammatory demyelination. Nat Commun. (2019) 10:3887. 10.1038/s41467-019-11638-3 31467299 PMC6715717

[103] Zhang SZ Wang QQ Yang QQ Gu HY Yin YQ Li YD. NG2 glia regulate brain innate immunity via TGF-beta2/TGFBR2 axis. BMC Med. (2019) 17:204. 10.1186/s12916-019-1439-x 31727112 PMC6857135

[104] Antel JP Lin YH Cui QL Pernin F Kennedy TE Ludwin SK. Immunology of oligodendrocyte precursor cells *in vivo* and *in vitro*. J Neuroimmunol. (2019) 331:28–35. 10.1016/j.jneuroim.2018.03.006 29566973

[105] Falcao AM van Bruggen D Marques S Meijer M Jakel S Agirre E. Disease-specific oligodendrocyte lineage cells arise in multiple sclerosis. Nat Med. (2018) 24:1837–44. 10.1038/s41591-018-0236-y 30420755 PMC6544508

[106] Bsibsi M Ravid R Gveric D van Noort JM. Broad expression of toll-like receptors in the human central nervous system. J Neuropathol Exp Neurol. (2002) 61:1013–21. 10.1093/jnen/61.11.1013 12430718

[107] Frederiksen HR Haukedal H Freude K. Cell type specific expression of toll-like receptors in human brains and implications in Alzheimer's disease. Biomed Res Int. (2019) 2019:7420189. 10.1155/2019/7420189 31396533 PMC6668540

[108] Bradl M Lassmann H. Oligodendrocytes: biology and pathology. Acta Neuropathol. (2010) 119:37–53. 10.1007/s00401-009-0601-5 19847447 PMC2799635

[109] Blank T Prinz M. NF-kappaB signaling regulates myelination in the CNS. Front Mol Neurosci. (2014) 7:47. 10.3389/fnmol.2014.00047 24904273 PMC4033361

[110] Karin M Lin A. NF-kappaB at the crossroads of life and death. Nat Immunol. (2002) 3:221–7. 10.1038/ni0302-221 11875461

[111] Rittchen S Boyd A Burns A Park J Fahmy TM Metcalfe S. Myelin repair *in vivo* is increased by targeting oligodendrocyte precursor cells with nanoparticles encapsulating leukaemia inhibitory factor (LIF). Biomaterials. (2015) 56:78–85. 10.1016/j.biomaterials.2015.03.044 25934281 PMC4429967

[112] Tsiperson V Huang Y Bagayogo I Song Y VonDran MW DiCicco-Bloom E. Brain-derived neurotrophic factor deficiency restricts proliferation of oligodendrocyte progenitors following cuprizone-induced demyelination. ASN Neuro. (2015) 7:1759091414566878. 10.1177/1759091414566878 25586993 PMC4720179

[113] Dombrowski Y O'Hagan T Dittmer M Penalva R Mayoral SR Bankhead P. Regulatory T cells promote myelin regeneration in the central nervous system. Nat Neurosci. (2017) 20:674–80. 10.1038/nn.4528 28288125 PMC5409501

[114] Pennati A Nylen EA Duncan ID Galipeau J. Regulatory B cells normalize CNS myeloid cell content in a mouse model of multiple sclerosis and promote oligodendrogenesis and remyelination. J Neurosci. (2020) 40:5105–15. 10.1523/JNEUROSCI.2840-19.2020 32430295 PMC7314404

[115] Zeis T Enz L Schaeren-Wiemers N. The immunomodulatory oligodendrocyte. Brain Res. (2016) 1641:139–48. 10.1016/j.brainres.2015.09.021 26423932

[116] Gaffen SL Jain R Garg AV Cua DJ. The IL-23-IL-17 immune axis: from mechanisms to therapeutic testing. Nat Rev Immunol. (2014) 14:585–600. 10.1038/nri3707 25145755 PMC4281037

[117] Brambilla R. The contribution of astrocytes to the neuroinflammatory response in multiple sclerosis and experimental autoimmune encephalomyelitis. Acta Neuropathol. (2019) 137:757–83. 10.1007/s00401-019-01980-7 30847559 PMC6483860

[118] Hoftberger R Aboul-Enein F Brueck W Lucchinetti C Rodriguez M Schmidbauer M. Expression of major histocompatibility complex class I molecules on the different cell types in multiple sclerosis lesions. Brain Pathol. (2004) 14:43–50. 10.1111/j.1750-3639.2004.tb00496.x 14997936 PMC8095881

[119] Fischer MT Sharma R Lim JL Haider L Frischer JM Drexhage J. NADPH oxidase expression in active multiple sclerosis lesions in relation to oxidative tissue damage and mitochondrial injury. Brain. (2012) 135:886–99. 10.1093/brain/aws012 22366799 PMC3286337

[120] Pekny M Pekna M. Astrocyte reactivity and reactive astrogliosis: costs and benefits. Physiol Rev. (2014) 94:1077–98. 10.1152/physrev.00041.2013 25287860

[121] Sirko S Behrendt G Johansson PA Tripathi P Costa M Bek S. Reactive glia in the injured brain acquire stem cell properties in response to sonic hedgehog. Cell Stem Cell. (2013) 12:426–39. 10.1016/j.stem.2013.01.019 23561443

[122] Anderson MA Burda JE Ren Y Ao Y O'Shea TM Kawaguchi R. Astrocyte scar formation aids central nervous system axon regeneration. Nature. (2016) 532:195–200. 10.1038/nature17623 27027288 PMC5243141

[123] Machado-Santos J Saji E Troscher AR Paunovic M Liblau R Gabriely G. The compartmentalized inflammatory response in the multiple sclerosis brain is composed of tissue-resident CD8+ T lymphocytes and B cells. Brain. (2018) 141:2066–82. 10.1093/brain/awy151 29873694 PMC6022681

[124] Haindl MT Kock U Zeitelhofer-Adzemovic M Fazekas F Hochmeister S. The formation of a glial scar does not prohibit remyelination in an animal model of multiple sclerosis. Glia. (2019) 67:467–81. 10.1002/glia.23556 30484905 PMC6588096

[125] Cho KS Yang L Lu B Feng Ma H Huang X Pekny M. Re-establishing the regenerative potential of central nervous system axons in postnatal mice. J Cell Sci. (2005) 118:863–72. 10.1242/jcs.01658 15731004 PMC1351228

[126] Li Q Barres BA. Microglia and macrophages in brain homeostasis and disease. Nat Rev Immunol. (2018) 18:225–42. 10.1038/nri.2017.125 29151590

[127] Seitz S Clarke P Tyler KL. Pharmacologic depletion of microglia increases viral load in the brain and enhances mortality in murine models of flavivirus-induced encephalitis. J Virol. (2018) 92:e00525–18. 10.1128/JVI.00525-18 29899084 PMC6069207

[128] Sanchez JMS DePaula-Silva AB Doty DJ Truong A Libbey JE Fujinami RS. Microglial cell depletion is fatal with low level picornavirus infection of the central nervous system. J Neurovirol. (2019) 25:415–21. 10.1007/s13365-019-00740-3 30859497 PMC6635090

[129] Heppner FL Ransohoff RM Becher B. Immune attack: the role of inflammation in Alzheimer disease. Nat Rev Neurosci. (2015) 16:358–72. 10.1038/nrn3880 25991443

[130] Condello C Yuan P Grutzendler J. Microglia-mediated neuroprotection, TREM2, and Alzheimer's disease: evidence from optical imaging. Biol Psychiatry. (2018) 83:377–87. 10.1016/j.biopsych.2017.10.007 29169609 PMC5767550

[131] Yuan P Condello C Keene CD Wang Y Bird TD Paul SM. TREM2 haplodeficiency in mice and humans impairs the microglia barrier function leading to decreased amyloid compaction and severe axonal dystrophy. Neuron. (2016) 92:252–64. 10.1016/j.neuron.2016.09.016 27710785

[132] Jay TR Hirsch AM Broihier ML Miller CM Neilson LE Ransohoff RM. Disease progression-dependent effects of TREM2 deficiency in a mouse model of alzheimer's disease. J Neurosci. (2017) 37, 637–47. 10.1523/JNEUROSCI.2110-16.2016 28100745 PMC5242410

[133] Heneka MT Kummer MP Stutz A Delekate A Schwartz S Vieira-Saecker A. NLRP3 is activated in Alzheimer's disease and contributes to pathology in APP/PS1 mice. Nature. (2013) 493:674–8. 10.1038/nature11729 23254930 PMC3812809

[134] Ulland TK Song WM Huang SC Ulrich JD Sergushichev A Beatty WL. TREM2 maintains microglial metabolic fitness in Alzheimer's disease. Cell. (2017) 170:649–63.e613. 10.1016/j.cell.2017.07.023 28802038 PMC5573224

[135] Wendeln AC Degenhardt K Kaurani L Gertig M Ulas T Jain G. Innate immune memory in the brain shapes neurological disease hallmarks. Nature, (2018) 556, 332–8. 10.1038/s41586-018-0023-4 29643512 PMC6038912

[136] Zhang CJ Jiang M Zhou H Liu W Wang C Kang Z. TLR-stimulated IRAKM activates caspase-8 inflammasome in microglia and promotes neuroinflammation. J Clin Invest. (2018) 128:5399–412. 10.1172/JCI121901 30372424 PMC6264724

[137] Nissen JC Thompson KK West BL Tsirka SE. Csf1R inhibition attenuates experimental autoimmune encephalomyelitis and promotes recovery. Exp Neurol. (2018) 307:24–36. 10.1016/j.expneurol.2018.05.021 29803827 PMC6380683

[138] Moreno MA Burns T Yao P Miers L Pleasure D Soulika AM. Therapeutic depletion of monocyte-derived cells protects from long-term axonal loss in experimental autoimmune encephalomyelitis. J Neuroimmunol. (2016) 290:36–46. 10.1016/j.jneuroim.2015.11.004 26711567

[139] Wolf Y Shemer A Levy-Efrati L Gross M Kim JS Engel A. Microglial MHC class II is dispensable for experimental autoimmune encephalomyelitis and cuprizone-induced demyelination. Eur J Immunol. (2018) 48:1308–18. 10.1002/eji.201847540 29697861

[140] Cabarrocas J Bauer J Piaggio E Liblau R Lassmann H. Effective and selective immune surveillance of the brain by MHC class I-restricted cytotoxic T lymphocytes. Eur J Immunol. (2003) 33:1174–82. 10.1002/eji.200323492 12731042

[141] Zia S Rawji KS Michaels NJ Burr M Kerr BJ Healy LM. Microglia diversity in health and multiple sclerosis. Front Immunol. (2020) 11:588021. 10.3389/fimmu.2020.588021 33240276 PMC7677361

[142] Miron VE Franklin RJ. Macrophages and CNS remyelination. J Neurochem. (2014) 130:165–71. 10.1111/jnc.12705 24601941

[143] Lytle JM Vicini S Wrathall JR. Phenotypic changes in NG2^+^ cells after spinal cord injury. J Neurotrauma, (2006) 23, 1726–38. 10.1089/neu.2006.23.1726 17184184

[144] Tatsumi K Takebayashi H Manabe T Tanaka KF Makinodan M Yamauchi T. Genetic fate mapping of Olig2 progenitors in the injured adult cerebral cortex reveals preferential differentiation into astrocytes. J Neurosci Res, (2008) 86, 3494–502. 10.1002/jnr.21862 18816798

[145] Hill RA Nishiyama A. NG2 cells (polydendrocytes): listeners to the neural network with diverse properties. Glia. (2014) 62:1195–210. 10.1002/glia.22664 24753030 PMC4282324

[146] Niu J Tsai HH Hoi KK Huang N Yu G Kim K. Aberrant oligodendroglial-vascular interactions disrupt the blood-brain barrier, triggering CNS inflammation. Nat Neurosci. (2019) 22:709–18. 10.1038/s41593-019-0369-4 30988524 PMC6486410

[147] Wennstrom M Janelidze S Bay-Richter C Minthon L Brundin L. Pro-inflammatory cytokines reduce the proliferation of NG2 cells and increase shedding of NG2 *in vivo* and *in vitro*. PLoS ONE. (2014) 9:e109387. 10.1371/journal.pone.0109387 25285951 PMC4186831

[148] International Multiple Sclerosis Genetics C. Multiple sclerosis genomic map implicates peripheral immune cells and microglia in susceptibility. Science. (2019) 365:aav7188. 10.1126/science.aav7188 31604244 PMC7241648

[149] Jakel S Agirre E Mendanha Falcao A van Bruggen D Lee KW Knuesel I. Altered human oligodendrocyte heterogeneity in multiple sclerosis. Nature. (2019) 566:543–7. 10.1038/s41586-019-0903-2 30747918 PMC6544546

[150] Schirmer L Velmeshev D Holmqvist S Kaufmann M Werneburg S Jung D. Neuronal vulnerability and multilineage diversity in multiple sclerosis. Nature. (2019) 573:75–82. 10.1038/s41586-019-1404-z 31316211 PMC6731122

[151] Chang A Nishiyama A Peterson J Prineas J Trapp BD. NG2-positive oligodendrocyte progenitor cells in adult human brain and multiple sclerosis lesions. J Neurosci. (2000) 20:6404–12. 10.1523/JNEUROSCI.20-17-06404.2000 10964946 PMC6772992

[152] Kuhlmann T Miron V Cui Q Wegner C Antel J Bruck W. Differentiation block of oligodendroglial progenitor cells as a cause for remyelination failure in chronic multiple sclerosis. Brain. (2008) 131:1749–58. 10.1093/brain/awn096 18515322

[153] Lassmann H van Horssen J Mahad D. Progressive multiple sclerosis: pathology and pathogenesis. Nat Rev Neurol. (2012) 8:647–56. 10.1038/nrneurol.2012.168 23007702

[154] Lisak RP Benjamins JA Nedelkoska L Barger JL Ragheb S Fan B. Secretory products of multiple sclerosis B cells are cytotoxic to oligodendroglia *in vitro*. J Neuroimmunol. (2012) 246:85–95. 10.1016/j.jneuroim.2012.02.015 22458983

[155] Watzlawik JO Wootla B Painter MM Warrington AE Rodriguez M. Cellular targets and mechanistic strategies of remyelination-promoting IgMs as part of the naturally occurring autoantibody repertoire. Expert Rev Neurother. (2013) 13:1017–29. 10.1586/14737175.2013.835601 24053345 PMC3909667

[156] Liddelow SA Barres BA. Reactive astrocytes: production, function, therapeutic potential. Immunity. (2017) 46:957–67. 10.1016/j.immuni.2017.06.006 28636962

[157] Wang C Zhang CJ Martin BN Bulek K Kang Z Zhao J. IL-17 induced NOTCH1 activation in oligodendrocyte progenitor cells enhances proliferation and inflammatory gene expression. Nat Commun. (2017) 8:15508. 10.1038/ncomms15508 28561022 PMC5460031

[158] Chew LJ King WC Kennedy A Gallo V. Interferon-gamma inhibits cell cycle exit in differentiating oligodendrocyte progenitor cells. Glia. (2005) 52:127–43. 10.1002/glia.20232 15920731

[159] Lin W Kemper A Dupree JL Harding HP Ron D Popko B. Interferon-gamma inhibits central nervous system remyelination through a process modulated by endoplasmic reticulum stress. Brain. (2006) 129:1306–18. 10.1093/brain/awl044 16504972

[160] Su Z Yuan Y Chen J Zhu Y Qiu Y Zhu F. Reactive astrocytes inhibit the survival and differentiation of oligodendrocyte precursor cells by secreted TNF-alpha. J Neurotrauma. (2011) 28:1089–100. 10.1089/neu.2010.1597 21309692

[161] Fernandez-Castaneda A Chappell MS Rosen DA Seki SM Beiter RM Johanson DM. The active contribution of OPCs to neuroinflammation is mediated by LRP1. Acta Neuropathol. (2020) 139:365–82. 10.1007/s00401-019-02073-1 31552482 PMC6994364

[162] Zhou Y Song WM Andhey PS Swain A Levy T Miller KR. Human and mouse single-nucleus transcriptomics reveal TREM2-dependent and TREM2-independent cellular responses in Alzheimer's disease. Nat Med. (2020) 26:131–42. 10.1038/s41591-019-0695-9 31932797 PMC6980793

[163] Mathys H Davila-Velderrain J Peng Z Gao F Mohammadi S Young JZ. Single-cell transcriptomic analysis of Alzheimer's disease. Nature. (2019) 570:332–37. 10.1038/s41586-019-1195-2 31042697 PMC6865822

[164] Srinivasan K Friedman B Etxeberria A Huntley M Brug M Foreman O. Alzheimer's patient brain myeloid cells exhibit enhanced aging and unique transcriptional activation. Cell Rep. (2019) 31:107843. 10.1016/j.celrep.2020.107843 32610143 PMC7422733

[165] Lin MT Beal MF. Mitochondrial dysfunction and oxidative stress in neurodegenerative diseases. Nature. (2006) 443:787–95. 10.1038/nature05292 17051205

[166] Rankin LC Artis D. Beyond host defense: emerging functions of the immune system in regulating complex tissue physiology. Cell. (2018) 173:554–67. 10.1016/j.cell.2018.03.013 29677509

[167] Louveau A Herz J Alme MN Salvador AF Dong MQ Viar KE. CNS lymphatic drainage and neuroinflammation are regulated by meningeal lymphatic vasculature. Nat Neurosci. (2018) 21:1380–91. 10.1038/s41593-018-0227-9 30224810 PMC6214619

[168] Kipnis J. Multifaceted interactions between adaptive immunity and the central nervous system. Science. (2016) 353:766–71. 10.1126/science.aag2638 27540163 PMC5590839

[169] Elmore MR Najafi AR Koike MA Dagher NN Spangenberg EE Rice RA. Colony-stimulating factor 1 receptor signaling is necessary for microglia viability, unmasking a microglia progenitor cell in the adult brain. Neuron. (2014) 82:380–97. 10.1016/j.neuron.2014.02.040 24742461 PMC4161285

[170] Filiano AJ Gadani SP Kipnis J. How and why do T cells and their derived cytokines affect the injured and healthy brain? Nat Rev Neurosci. (2017) 18:375–84. 10.1038/nrn.2017.39 28446786 PMC5823005

[171] Radjavi A Smirnov I Kipnis J. Brain antigen-reactive CD4+ T cells are sufficient to support learning behavior in mice with limited T cell repertoire. Brain Behav Immun. (2014) 35:58–63. 10.1016/j.bbi.2013.08.013 24012647 PMC3858511

[172] Kipnis J Cohen H Cardon M Ziv Y Schwartz M. T cell deficiency leads to cognitive dysfunction: implications for therapeutic vaccination for schizophrenia and other psychiatric conditions. Proc Natl Acad Sci USA. (2004) 101:8180–5. 10.1073/pnas.0402268101 15141078 PMC419577

[173] Brynskikh A Warren T Zhu J Kipnis J. Adaptive immunity affects learning behavior in mice. Brain Behav Immun. (2008) 22:861–9. 10.1016/j.bbi.2007.12.008 18249087

[174] Kipnis J Gadani S Derecki NC. Pro-cognitive properties of T cells. Nat Rev Immunol. (2012) 12:663–9. 10.1038/nri3280 22903149 PMC4032225

[175] Derecki NC Cardani AN Yang CH Quinnies KM Crihfield A Lynch KR. Regulation of learning and memory by meningeal immunity: a key role for IL-4. J Exp Med. (2010) 207:1067–80. 10.1084/jem.20091419 20439540 PMC2867291

[176] Brombacher TM Nono JK De Gouveia KS Makena N Darby M Womersley J. IL-13-mediated regulation of learning and memory. J Immunol. (2017) 198:2681–8. 10.4049/jimmunol.1601546 28202615

[177] Sotomayor-Sobrino MA Ochoa-Aguilar A Mendez-Cuesta LA Gomez-Acevedo C. Neuroimmunological interactions in stroke. Neurologia. (2019) 34:326–35. 10.1016/j.nrl.2016.08.003 27776957

[178] Moalem G Leibowitz-Amit R Yoles E Mor F Cohen IR Schwartz M. Autoimmune T cells protect neurons from secondary degeneration after central nervous system axotomy. Nat Med. (1999) 5:49–55. 10.1038/4734 9883839

[179] Walsh JT Hendrix S Boato F Smirnov I Zheng J Lukens JR. MHCII-independent CD4+ T cells protect injured CNS neurons via IL-4. J Clin Invest. (2015) 125:699–714. 10.1172/JCI76210 25607842 PMC4319416

[180] Lammert CR Frost EL Bolte AC Paysour MJ Shaw ME Bellinger CE. Cutting edge: critical roles for microbiota-mediated regulation of the immune system in a prenatal immune activation model of autism. J Immunol. (2018) 201:845–50. 10.4049/jimmunol.1701755 29967099 PMC6057827

[181] Shin Yim Y Park A Berrios J Lafourcade M Pascual LM Soares N. Reversing behavioural abnormalities in mice exposed to maternal inflammation. Nature. (2017) 549:482–7. 10.1038/nature23909 28902835 PMC5796433

[182] Korn T Bettelli E Oukka M Kuchroo VK. IL-17 and Th17 cells. Annu Rev Immunol. (2009) 27:485–517. 10.1146/annurev.immunol.021908.132710 19132915

[183] Rothhammer V Borucki DM Tjon EC Takenaka MC Chao CC Ardura-Fabregat A. Microglial control of astrocytes in response to microbial metabolites. Nature. (2018) 557:724–8. 10.1038/s41586-018-0119-x 29769726 PMC6422159

[184] Filiano AJ Xu Y Tustison NJ Marsh RL Baker W Smirnov I. Unexpected role of interferon-gamma in regulating neuronal connectivity and social behaviour. Nature. (2016) 535:425–9. 10.1038/nature18626 27409813 PMC4961620

[185] Dulken BW Buckley MT Navarro Negredo P Saligrama N Cayrol R Leeman DS. Single-cell analysis reveals T cell infiltration in old neurogenic niches. Nature. (2019) 571:205–10. 10.1038/s41586-019-1362-5 31270459 PMC7111535

[186] Garber C Soung A Vollmer LL Kanmogne M Last A Brown J. T cells promote microglia-mediated synaptic elimination and cognitive dysfunction during recovery from neuropathogenic flaviviruses. Nat Neurosci. (2019) 22:1276–88. 10.1038/s41593-019-0427-y 31235930 PMC6822175

[187] Dedoni S Olianas MC Ingianni A Onali P. Type I interferons impair BDNF-induced cell signaling and neurotrophic activity in differentiated human SH-SY5Y neuroblastoma cells and mouse primary cortical neurons. J Neurochem. (2012) 122:58–71. 10.1111/j.1471-4159.2012.07766.x 22533963

[188] West PK Viengkhou B Campbell IL Hofer MJ. Microglia responses to interleukin-6 and type I interferons in neuroinflammatory disease. Glia. (2019) 67:1821–41. 10.1002/glia.23634 31033014

[189] Reyes-Vazquez C Prieto-Gomez B Dafny N. Interferon modulates central nervous system function. Brain Res. (2012) 1442:76–89. 10.1016/j.brainres.2011.09.061 22322149

[190] Kipnis J. Immune system: the “seventh sense”. J Exp Med. (2018) 215:397–8. 10.1084/jem.20172295 29339443 PMC5789422

[191] Gadani SP Smirnov I Smith AT Overall CC Kipnis J. Characterization of meningeal type 2 innate lymphocytes and their response to CNS injury. J Exp Med. (2017) 214:285–96. 10.1084/jem.20161982 27994070 PMC5294864

[192] Klose CSN Mahlakoiv T Moeller JB Rankin LC Flamar AL Kabata H. The neuropeptide neuromedin U stimulates innate lymphoid cells and type 2 inflammation. Nature. (2017) 549:282–6. 10.1038/nature23676 28869965 PMC6066372

[193] Gourraud PA Henry RG Cree BA Crane JC Lizee A Olson MP. Precision medicine in chronic disease management: the multiple sclerosis BioScreen. Ann Neurol. (2014) 76:633–42. 10.1002/ana.24282 25263997 PMC4214886

[194] Steinman L. Immunology of relapse and remission in multiple sclerosis. Annu Rev Immunol. (2014) 32:257–81. 10.1146/annurev-immunol-032713-120227 24438352

[195] Gossman W Ehsan M Xixis KL. Multiple Sclerosis. Treasure Island, FL: StatPearls (2020).

[196] Karpus WJ. Cytokines and chemokines in the pathogenesis of experimental autoimmune encephalomyelitis. J Immunol. (2020) 204:316–26. 10.4049/jimmunol.1900914 31907274

[197] Matthews PM Block VJ Leocani L. E-health and multiple sclerosis. Curr Opin Neurol. (2020) 33:271–6. 10.1097/WCO.0000000000000823 32324706

[198] Wiedrick J Meza-Romero R Gerstner G Seifert H Chaudhary P Headrick A. Sex differences in EAE reveal common and distinct cellular and molecular components. Cell Immunol. (2021) 359:104242. 10.1016/j.cellimm.2020.104242 33190849 PMC7770093

[199] Hemmer B Kerschensteiner M Korn T. Role of the innate and adaptive immune responses in the course of multiple sclerosis. Lancet Neurol. (2015) 14:406–19. 10.1016/S1474-4422(14)70305-9 25792099

[200] Simmons SB Pierson ER Lee SY Goverman JM. Modeling the heterogeneity of multiple sclerosis in animals. Trends Immunol. (2013) 34:410–22. 10.1016/j.it.2013.04.006 23707039 PMC3752929

[201] Aube B Levesque SA Pare A Chamma E Kebir H Gorina R. Neutrophils mediate blood-spinal cord barrier disruption in demyelinating neuroinflammatory diseases. J Immunol. (2014) 193:2438–54. 10.4049/jimmunol.1400401 25049355

[202] Croxford AL Spath S Becher B. GM-CSF in neuroinflammation: licensing myeloid cells for tissue damage. Trends Immunol. (2015) 36:651–62. 10.1016/j.it.2015.08.004 26431942

[203] Wekerle H. B cells in multiple sclerosis. Autoimmunity. (2017) 50:57–60. 10.1080/08916934.2017.1281914 28166681

[204] Mimpen M Smolders J Hupperts R Damoiseaux J. Natural killer cells in multiple sclerosis: a review. Immunol Lett. (2020) 222:1–11. 10.1016/j.imlet.2020.02.012 32113900

[205] Lassmann H Bradl M. Multiple sclerosis: experimental models and reality. Acta Neuropathol. (2017) 133:223–44. 10.1007/s00401-016-1631-4 27766432 PMC5250666

[206] Babbe H Roers A Waisman A Lassmann H Goebels N Hohlfeld R. Clonal expansions of CD8(+) T cells dominate the T cell infiltrate in active multiple sclerosis lesions as shown by micromanipulation and single cell polymerase chain reaction. J Exp Med. (2000) 192:393–404. 10.1084/jem.192.3.393 10934227 PMC2193223

[207] Bianchini E De Biasi S Simone AM Ferraro D Sola P Cossarizza A. Invariant natural killer T cells and mucosal-associated invariant T cells in multiple sclerosis. Immunol Lett. (2017) 183:1–7. 10.1016/j.imlet.2017.01.009 28119072

[208] Gris D Ye Z Iocca HA Wen H Craven RR Gris P. NLRP3 plays a critical role in the development of experimental autoimmune encephalomyelitis by mediating Th1 and Th17 responses. J Immunol. (2010) 185:974–81. 10.4049/jimmunol.0904145 20574004 PMC3593010

[209] Bruck W Stadelmann C. Inflammation and degeneration in multiple sclerosis. Neurol Sci. (2003) 24(Suppl. 5):S265–7. 10.1007/s10072-003-0170-7 14652785

[210] Friese MA Schattling B Fugger L. Mechanisms of neurodegeneration and axonal dysfunction in multiple sclerosis. Nat Rev Neurol. (2014) 10:22538. 10.1038/nrneurol.2014.37 24638138

[211] Olivares-Villagomez D Wang Y Lafaille JJ. Regulatory CD4(+) T cells expressing endogenous T cell receptor chains protect myelin basic protein-specific transgenic mice from spontaneous autoimmune encephalomyelitis. J Exp Med. (1998) 188:1883–94. 10.1084/jem.188.10.1883 9815266 PMC2212402

[212] Van de Keere F Tonegawa S. CD4(+) T cells prevent spontaneous experimental autoimmune encephalomyelitis in anti-myelin basic protein T cell receptor transgenic mice. J Exp Med. (1998) 188:1875–82. 10.1084/jem.188.10.1875 9815265 PMC2212404

[213] Boitard C Yasunami R Dardenne M Bach JF. T cell-mediated inhibition of the transfer of autoimmune diabetes in NOD mice. J Exp Med. (1989) 169:1669–80. 10.1084/jem.169.5.1669 2523954 PMC2189316

[214] Matejuk A Buenafe AC Dwyer J Ito A Silverman M Zamora A. Endogenous CD4+BV8S2- T cells from TG BV8S2+ donors confer complete protection against spontaneous experimental encephalomyelitis (Sp-EAE) in TCR transgenic, RAG-/- mice. J Neurosci Res. (2003) 71:89–103. 10.1002/jnr.10450 12478617

[215] Vandenbark AA Offner H. Critical evaluation of regulatory T cells in autoimmunity: are the most potent regulatory specificities being ignored? Immunology. (2008) 125:1–13. 10.1111/j.1365-2567.2008.02900.x 18798915 PMC2526254

[216] Mann MK Maresz K Shriver LP Tan Y Dittel BN. B cell regulation of CD4+CD25+ T regulatory cells and IL-10 via B7 is essential for recovery from experimental autoimmune encephalomyelitis. J Immunol. (2007) 178:3447–56. 10.4049/jimmunol.178.6.3447 17339439

[217] Engelhardt B Carare RO Bechmann I Flugel A Laman JD Weller RO. Vascular, glial, and lymphatic immune gateways of the central nervous system. Acta Neuropathol. (2016) 132:317–38. 10.1007/s00401-016-1606-5 27522506 PMC4992028

[218] Beers DR Henkel JS Zhao W Wang J Appel SH. CD4+ T cells support glial neuroprotection, slow disease progression, and modify glial morphology in an animal model of inherited ALS. Proc Natl Acad Sci USA. (2008) 105:15558–63. 10.1073/pnas.0807419105 18809917 PMC2547419

[219] Marsh SE Abud EM Lakatos A Karimzadeh A Yeung ST Davtyan H. The adaptive immune system restrains Alzheimer's disease pathogenesis by modulating microglial function. Proc Natl Acad Sci USA. (2016) 113:E1316–25. 10.1073/pnas.1525466113 26884167 PMC4780638

[220] Serpe CJ Kohm AP Huppenbauer CB Sanders VM Jones KJ. Exacerbation of facial motoneuron loss after facial nerve transection in severe combined immunodeficient (scid) mice. J Neurosci. (1999)19:RC7. 10341268 10.1523/JNEUROSCI.19-11-j0004.1999 PMC6782611

[221] Gadani SP Walsh JT Smirnov I Zheng J Kipnis J. The glia-derived alarmin IL-33 orchestrates the immune response and promotes recovery following CNS injury. Neuron. (2015) 85:703–9. 10.1016/j.neuron.2015.01.013 25661185 PMC12064979

